# A robust spectral angle index for remotely assessing soybean canopy chlorophyll content in different growing stages

**DOI:** 10.1186/s13007-020-00643-z

**Published:** 2020-07-31

**Authors:** Jibo Yue, Haikuan Feng, Qingjiu Tian, Chengquan Zhou

**Affiliations:** 1 Key Laboratory of Quantitative Remote Sensing in Agriculture of Ministry of Agriculture, Beijing Research Center for Information Technology in Agriculture, Beijing, 100097 China; 2 International Institute for Earth System Science, Nanjing University, Nanjing, 210023 China; 3 Institute of Agricultural Equipment, Zhejiang Academy of Agricultural Sciences (ZAAS), Hangzhou, 310000 China

**Keywords:** Angle index, Spectral vegetation indices, UAV remote sensing, Soybean

## Abstract

**Background:**

Timely and accurate estimates of canopy chlorophyll (Chl) a and b content are crucial for crop growth monitoring and agricultural management. Crop canopy reflectance depends on many factors, which can be divided into the following categories: (i) leaf effects (e.g., leaf pigments), (ii) canopy effects (e.g., Leaf Area Index [LAI]), and (iii) soil background reflectance (e.g., soil reflectance). The estimation of leaf variables, such as Chl contents, from reflectance at the canopy scale is usually less accurate than that at the leaf scale. In this study, we propose a Visible and Near-infrared (NIR) Angle Index (VNAI) to estimate the Chl content of soybean canopy, and soybean canopy Chl maps are produced using visible and NIR unmanned aerial vehicle (UAV) remote sensing images. The VNAI is insensitive to LAI and can be used for the multi-stage estimation of crop canopy Chl content.

**Results:**

Eleven previously used vegetation indices (VIs) (e.g., Pigment-specific Normalized Difference Index) were selected for performance comparison. The results showed that (i) most previously used Chl VIs were significantly correlated with LAI, and the proposed VNAI was more sensitive to Chl content than LAI; (ii) the VNAI-based estimates of Chl content were more accurate than those based on the other investigated VIs using (1) simulated, (2) real (field), and (3) real (UAV) datasets.

**Conclusions:**

Most previously used Chl VIs were significantly correlated with LAI whereas the proposed VNAI was more sensitive to Chl content than to LAI, indicating that the VNAI may be more strongly correlated with Chl content than these previously used VIs. Multi-stage estimations of the Chl content of cropland obtained using the VNAI and broadband remote sensing images may help to obtain Chl maps with high temporal and spatial resolution.

## Background

Chlorophyll (Chl) a and b are the most important pigments for photosynthesis in green vegetation [1–4]. Leaf and canopy Chl content can directly reflect the nutrient status of plant stress and senescence status since (i) nitrogen is a component of Chl and (ii) nitrogen is related to many physiological stresses of crops [5–10]. Timely and accurate estimates of canopy Chl concentrations are crucial in crop growth monitoring and agricultural management. Prior to the establishment of remote sensing techniques, the ability to perform large-scale crop Chl surveys was limited, since traditional manual methods for the measurement of crop Chl contents are inefficient, costly, and cannot provide crop Chl maps over large areas.

Optical remote sensing can capture the surface radiation that is emitted from the Earth at visible to near-infrared (NIR) and short-wave infrared (SWIR) wavelengths. Visible and NIR bands are crucial for estimating crop parameters [11]. The crop spectral reflectance in the blue and red bands is lower than that in other optical bands as a result of Chl absorption [11]. Additionally, crop spectral reflectance in the NIR region is much higher than that in the visible band [12]. The features described above can be detected by using broadband satellite remote sensing, and accordingly many broadband satellite-based remote sensing vegetation indices (VIs) have been developed to monitor vegetation parameters.

Green vegetation leaf reflectance primarily depends on several leaf parameters, such as the internal structural parameters of the leaf mesophyll, pigment content (e.g., Chl, carotenoid, and anthocyanin), leaf water content, and leaf dry matter content [13–15]. The absorption features of chlorophylls, carotenoids, and anthocyanins are located in the visible bands [16–18]. Water absorbs radiation from the visible to the SWIR band; however, leaf water content is mostly affected by leaf reflectance in the NIR and SWIR bands [16–18]. Leaf dry matter content influences multiple intercellular scattering and affects the canopy reflectance in the NIR bands [16]. Additionally, the leaf reflectance properties in the visible bands have been found to be dependent on Chl content according to many global sensitivity analyses based on the Properties Optique Spectrales des Feuilles (PROSPECT) model [19].

Many field-, laboratory-, and PROSPECT-based estimates of Chl content have been successfully carried out in recent years. These studies can be divided into the following categories: (i) radiative transfer models (RTMs), (ii) VIs, (iii) empirical regression, and (iv) synergistic methods. RTMs (e.g., PROSPECT, a combination of PROSPECT and the Scattering by Arbitrarily Inclined Leaves (SAIL) model [PROSAIL], Invertible Forest Reflectance Model [INFORM]) are founded on physical principles and can be used to simulate leaf optical reflectance [13, 14]. Spectral VIs have been widely used to estimate Chl, such as the Pigment-specific Normalized Difference Index [20], the Blue Red Pigment Index [21], and the Normalized Total Pigment to Chl Index [22]. Empirical regression methods (e.g., partial least squares regression [PLSR], artificial neural networks, and support vector machine regression [SVR]) can be used to determine direct relationships between spectral features (spectral reflectance or pigment VIs) and Chl content by using a large number of ground measurements [23, 24]. Synergistic methods estimate Chl content by combining multiple techniques (e.g., INFORM + lookup table (LUT) method [25], PROSAIL + LUT method [26, 27], VIs + SVR [28, 29], VIs + PLSR [30], PROSAIL + VIs [31]).

Crop canopy reflectance depends on many factors, which can be divided into the following categories: (i) leaf effects (e.g., leaf pigments and leaf water content), (ii) canopy biophysical effects (e.g., Leaf Area Index [LAI], leaf inclination angle), and (iii) soil background reflectance (e.g., soil moisture and soil reflectance) [32–34]. The soil background and canopy effects cause a series of problems in the canopy-scale estimation of leaf parameters. At the local-scale, some factors can be assumed to be negligible due to their stable within-crop variation [16]. The PROSAIL model can be used to analyze the spectral reflectance of vegetation canopy at wavelengths of 400–2500 nm [34, 35]. A global sensitivity analysis using the PROSAIL model showed that LAI is a key variable that governs the crop canopy reflectance properties over the entire spectrum [32]. The absorption features of pigments are located in the visible bands [36–39], and therefore, the estimation of leaf variables (e.g., Chl content) based on reflectance data is usually less accurate at the canopy-scale than at the leaf-scale as a result of canopy effects [40–44].

Previous studies have developed hyperspectral techniques for the estimation of crop canopy Chl content, which are less sensitive to canopy effects. Four examples of such methods are: (i) Chl VIs based on optimum band combinations [45]; (ii) band-depth analysis techniques [46, 47]; (iii) continuous wavelet transform techniques [48, 49]; and (iv) red-edge-based techniques [50–54]. By testing all available band combinations, optimum band combination methods return VIs with the highest accuracy of all available band combinations [45]. Usually, optimum band combination methods achieve high accuracy when mapping crop Chl content at the local scale. Due to the deepening and widening of the red absorption region with increasing Chl content, band-depth analysis techniques can be used to quantify the canopy Chl content based on visible and NIR hyperspectral reflectance [46, 47]. Wavelet transform techniques are effective tools for signal analysis that return the wavelet coefficients of hyperspectral reflectance, which can be used as the modeling variables to estimate Chl content [48, 49]. The “red edge” refers to the rapid change in the hyperspectral reflectance of vegetation that occurs in the NIR bands as vegetation grows [50–54]. The red edge arises due to (a) the Chl absorption in the visible bands and (b) the high hyperspectral reflectivity of vegetation in the NIR region. In recent years, several promising red-edge-based Chl VIs have been developed for the estimation of the Chl content of field canopies [50, 55, 56]. For instance, Daughtry et al. [55] showed that the ratio of the Red-edge-based Transformed Chlorophyll Absorption Reflectance Index (TCARI) and the Optimized Soil-adjusted VI (OSAVI) was linearly related to leaf Chl content in a variety of vegetation cover and soil background types. Additionally, Barnes et al. [57] used the Normalized Difference Red-edge Index (NDRE: (*R*_790_−*R*_720_)/(*R*_790_ + *R*_720_)) to quantify canopy Chl content. Their results suggested that the index can be used to estimate Chl content. Moreover, the results of Gitelson et al. [50] also indicated that the ratio of two red-edge bands (705 nm and 783 nm) (Red-edge Chlorophyll Index [CI(red edge)]: (*R*_783_/*R*_705_) − 1) can be used to estimate the Chl content of vegetation.

However, unlike Chl estimation methods based on widely used cost-free satellite broadband remote sensing data (e.g., Landsat Thematic Mapper [TM]/Enhanced Thematic Mapper Plus [ETM +]/Operational Land Imager [OLI]), Chl estimation methods based on narrowband hyperspectral and red-edge remote sensing data are limited due to the scarcity of sensors [58, 59]. As mentioned before, most high-performance hyperspectral-based and red-edge-based remote sensing techniques cannot be applied to broadband remote sensing. Thus, it is important to establish a broadband remote sensing Chl VI which is less sensitive to canopy effects.

This work aimed to develop a broadband remote sensing Chl VI for the multi-stage estimation of crop canopy Chl content that is insensitive to LAI. We propose a Visible and Near-infrared Angle Index (VNAI) to obtain estimates of soybean canopy Chl content and produce soybean canopy Chl maps by using broadband visible and NIR unmanned aerial vehicle (UAV) remote sensing images. The effects of LAI on canopy spectral were mitigated by using the VNAI. Our study evaluated (i) the response of the proposed VNAI and several existing Chl VIs to Chl content and LAI, and (ii) the performance of the estimation of Chl content using soybean canopy spectral data and soybean canopy Chl mapping using UAV-based remote sensing images.

## Material

### Study area and field experimental design

**Fig. 1:**
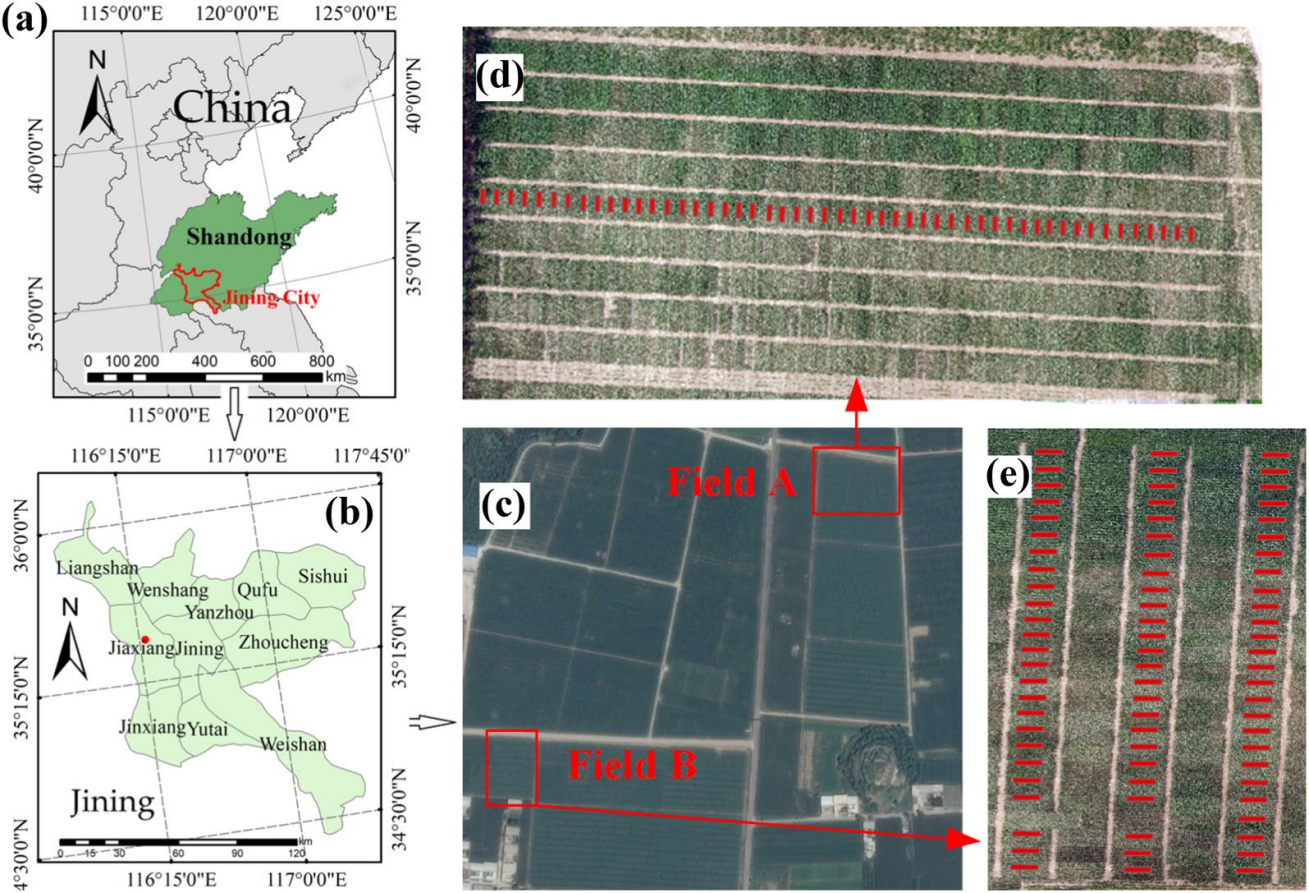
The location of the study area and field experimental design: **a** location of Jining City in China, **b** location of Jiaxiang County in Jining City, **c** experimental field, **d** experimental design of Field A, and **e** experimental design of Field B

The study area was located in Jiaxiang County (Fig. 1a and b, 35.4324° N, 116.3675° E), Jining City, Shandong Province, China. Jiaxiang County has an average altitude, temperature, and rainfall of 35 m a.s.l., 13.9 °C, and 701 mm, respectively. Winter wheat (September to June) and soybean and maize (June to September) are the main crops that are planted in this county. Two fields were selected for the field experiment (Fig. 1). The field experimental design is shown in Fig. 1. A total of 127 plots (Field A: 51; Field B: 76) were selected for field measurement. The soybean sowing date was 13 June 2015, the planting density was approximately 190,000 plants/ha, the row spacing was 50 cm, and the plot size was approximately 5 m × 2.5 m. Soybean canopy spectral reflectance, leaf Chl content, and UAV-based spectral measurements were conducted at five growth stages, namely flowering (S1), early podding (S2), later podding (S3), grain filling (S4), and harvest (S5). Field- and UAV-based canopy spectral reflectance was measured using a spectrometer (FieldSpec 3; Analytical Spectral Devices, Boulder, CO, USA) and a snapshot spectrometer (UHD 185; Cubert GmbH, Ulm, Baden-Württemberg, Germany). Leaf Chl content was measured using a Dualex scientific portable sensor (Dualex 4; Force-A, Orsay, France [60]).

### UAV-based acquisition of canopy spectral data

In this work, UAV-based canopy spectral measurements were made in Field A before the collection of field Chl and spectral data. The spectral measurements were made between 11:00 A.M. and 2:00 P.M. The UAV flying height was set to approximately 50 m and all UAV flights were conducted under cloud-free conditions. The field spectral images were collected using a snapshot hyperspectral sensor (UHD 185, see Table 1) mounted on an eight-armed DJI S-1000 UAV (Dajiang Innovation, Sham Chun, China). The operating range of the UHD 185 spans the visible to the NIR (wavelengths of 450–950 nm; Table 1). The parameters of the UAV and the UHD 185 sensor are detailed in Table 1.

**Table 1:** The parameters of the unmanned aerial vehicle (UAV), UHD 185 hyperspectral imaging sensor, and FieldSpec 3 field spectrometer that were used in this study

UAV		Sensors and parameters		
		Types	UHD185^a^	FieldSpec 3^b^
Name	DJI S-1000	Field of view	19°	25°
Flying height	50 m	Working height	50 m above ground	0.5 m above canopy
Flying speed	About 8 m/s	Spectral information	450–950 nm	350–2500 nm
Takeoff weight	6–11 kg	Original spectral resolution	8 nm @ 532 nm	3 nm @ 700 nm; 8.5 nm @ 1400 nm; 6.5 nm @ 2100 nm
Working time	About 30 min	Data spectral resolution	4 nm	1 nm

^a^The UHD 185 was mounted on the UAV

^b^The FieldSpec 3 was used for field canopy spectral measurements

For the collection of UAV-based hyperspectral images, four steps were taken:

(i) First, before each flight, the UHD 185 spectrometer was initialized in factory settings using the Cubert-Pilot software (Cubert GmbH), and then a white reference was taken from a reflector which can be considered as a Lambert reflector (spectral reflectance = 1);
(ii) Then, an opaque lens cap was used to block the light from entering the spectrometer, which can be considered as non-reflecting (spectral reflectance = 0). Subsequently, the UHD 185 spectrometer was calibrated and the exposure time was determined using the Cubert-Pilot software;
(iii) The forward and side overlaps of the hyperspectral images were set to 70%. The hyperspectral images were collected using Cubert-Pilot software onboard the UAV; besides, the position and orientation system (POS) data was also recorded by using an inertial measurement unit. The field soybean canopy reflectance data were recorded in 125-band hyperspectral images (data spectral resolution: 4 nm);
(iv) Finally, the hyperspectral images were stitched together using the Agisoft PhotoScan software (Agisoft LLC, St. Petersburg, Russia) to produce hyperspectral digital orthophoto maps (DOMs) of the experimental field.

Remote sensing images acquired from high-altitude platforms typically require atmospheric correction. However, in this study, the hyperspectral images were obtained under stable light conditions at an altitude of 50 m, and therefore atmospheric correction was not required. The accuracy of hyperspectral DOMs produced using data acquired at low altitude using a UHD-185 was verified in our previous study [61]. The ground spatial resolution of the hyperspectral DOMs is about 3 cm. After image collection and the stitching process, a total of four DOMs were produced for the experimental field. For the extraction of field canopy spectra, two steps were taken:

(i) Hyperspectral DOMs were imported into the ENVI software (Harris Geospatial Solutions, Boulder, CO, USA) and then the regions of interest (ROIs) in all soybean plots were manually delineated;
(ii) The field canopy spectra of each plot were extracted from the DOMs using the ROI tools in the ENVI software.

### Field-based canopy spectra

After the UAV flight, field soybean canopy spectral reflectance measurements were conducted under cloud-free conditions. Chl and soybean canopy spectral data were collected at the same sampling position in each soybean plot. We used a spectrometer (FieldSpec 3, see Table 1 for spectral parameters) to collect the soybean canopy hyperspectral reflectance at the center of each soybean plot. For the field canopy hyperspectral measurements, four steps were taken:

(i) First, to avoid shadow effects, the optical fiber of the FieldSpec 3 was located 0.2 m above the white reference panel and along rays of sunlight;
(ii) Subsequently, the instrument was optimized to eliminate the dark current effect and then the white reference was taken;
(iii) The optical fiber was located 0.5 m above the crop canopy and along rays of sunlight and then the canopy hyperspectral measurement was made;
(iv) The white reference panel was remeasured, and step (ii) was repeated if the spectral reflectance value was higher than 1.05 or lower than 0.95.

Since the FieldSpec 3 has a 25° field of view, the hyperspectral reflectance can be collected from a circular area of canopy with a diameter of 22.5 cm. For each plot, hyperspectral reflectance measurements were repeated 10 times and the average value was taken as the canopy spectrum of the plot.

### Field measurement of leaf and canopy Chl

After the measurement of soybean canopy reflectance, measurements of soybean canopy leaf Chl content were conducted. The soybean canopy leaves were selected from the spectral collection area (i.e., a circle of canopy with a diameter of 22.5 cm). The soybean leaf Chl content was measured using a Dualex 4 scientific portable sensor and the collected dataset was named as the Chl-D dataset. The Chl content measured with the Dualex 4 was in the range of 5–80 µg/cm^2^. The Dualex instrument enables the rapid measurement of leaf Chl content under field conditions. The measurements were performed at the first and second uppermost leaves. Five measurements of each leaf were performed at the center of each soybean plot. In this work, we focused on the leaves at the top of the canopy since these contribute the most to the canopy reflectance. After the collection of leaf Chl data, the average values were recorded as the canopy Chl content of each soybean plot. The results are given in Table 2. As shown in the table, from 29 July 2015 to 28 September 2015, the leaf Chl content of fields A and B first increases (S1–S3) and then decreases (S4–S5).

**Table 2:** The measured values of chlorophyll (Chl) content (Dualex units) in the study area

Field	Stage	UAV DOMs	Date	Number of plots	Measured Chl content				
					Min	Max	Mean	SD	COV
Field A	S1	√	29 July 2015	51	25.41	34.32	30.24	1.88	6.2%
	S2	√	13 August 2015	51	28.07	38.43	32.86	2.45	7.4%
	S3	√	31 August 2015	51	31.18	46.18	39.79	3.03	7.6%
	S4	√	17 September 2015	42	7.78	33.34	20.07	5.28	26.3%
	S5	–	28 September 2015	11	10.59	27.89	19.77	5.42	27.4%
Field B	S1	–	29 July 2015	76	23.49	40.80	30.57	2.91	9.5%
	S2	–	13 August 2015	76	23.05	38.10	31.25	3.48	11.2%
	S3	–	31 August 2015	76	30.65	48.64	39.94	4.59	11.5%
	S4	–	17 September 2015	76	17.09	45.93	31.7	6.62	20.9%
	S5	–	28 September 2015	66	9.57	50.57	27.44	8.76	31.9%

DOM: digital orthophoto map. SD: standard deviation. COV: coefficient of variation. The soybean leaf Chl content was measured using a Dualex 4 scientific portable sensor. The Chl content measured by the Dualex 4 is given in µg/cm^2^ in the range of 5–80 µg/cm^2^ (see https://www.force-a.com/products/dualex). However, in many studies, the unit of Chl measured by the Dualex 4 is marked as “Dualex units” (see [62–64]); thus, we also used “Dualex units” instead of µg/cm^2^ for the soybean leaf Chl content dataset. Min, Max, and Mean represent the minimum, maximum, and average Chl contents, respectively. The number of sampling plots is different since some plots containing early-maturing varieties were harvested during stages S4 and S5. “√” indicates that UAV-based canopy spectral images were collected and “-” indicates that UAV-based canopy spectral images were not collected. Most of the plant plots in Field A were harvested during stages S4 and S5. This is due to the fact that early- and late-maturing varieties were planted in Field A and Field B, respectively

### PROSAIL-based canopy spectral

A PROSAIL RTM was used to analyze the spectral reflectance of the vegetation canopy at wavelengths of 400 to 2500 nm [34, 35]. The PROSAIL model is a combination of the PROSPECT [14] and SAIL [65] models, and its inputs include several leaf (see PROSPECT parameters in Table 3), canopy, and soil parameters (see SAIL parameters in Table 3), which have been previously described in [32, 35]. The PROSAIL RTM has been widely used to analyze the effects of leaf, canopy, and soil parameters on canopy reflectance.

**Table 3:** The parameter settings of the PROSAIL model (a combination of the Properties Optique Spectrales des Feuilles [PROSPECT] model and the Scattering by Arbitrarily Inclined Leaves [SAIL] model)

Type	Parameter	Values/ranges
Leaf parameters	Leaf structure index (N)	1.5
	Chlorophyll content (Cab)	(a) 10:1:40 μg/cm^2^ (b) 20:1:45 μg/cm^2^ (c) 25:1:50 μg/cm^2^
	Carotenoid content (Car)	0 μg/cm^2^
	Brown pigments (Cbrown)	0 μg/cm^2^
	Dry matter content (Cm)	0.01 g/cm^2^
	Equivalent water thickness (Cw)	0.02 cm
Canopy structure and observation	Hot spot (hspot)	0.5
	Solar zenith angle (tts)	20*°*
	Observer zenith angle (tto)	0*°*
	Azimuth (psi)	90*°*
	Average leaf inclination angle (ALA)	60*°*
	Leaf area index (LAI)	(a) 2:0.5:4 m^2^/m^2^ (b) 4.5:0.5:6 m^2^/m^2^ (c) 6.5:0.5:8 m^2^/m^2^
	Soil moisture factor (psoil)	0

The chlorophyll content dataset is labeled as “Chl-Cab” to correspond with the labeling of the field-based measurements of Chl content (Chl-D)

The global sensitivity analysis of the PROSAIL model showed that LAI and Chl content are the two most important factors which control the crop canopy reflectance in the visible and NIR bands [32]. More specifically, LAI is a key variable that governs the crop canopy reflectance properties over the entire spectrum [32], while Chl has a large effect on crop canopy reflectance in the visible bands [36–39]. In this work, the LAI and Chl content parameters of PROSAIL were special settings to represent the reality of cropland.

We measured the soybean LAI (see Additional file 1: Fig. S2) and Chl content from stages S1 to S5. Then, the field-measured soybean LAI and Chl content were used as the LAI and Chl content parameters of PROSAIL (see Additional file 1: Fig. S2, (a) Chl-Cab (parameter Cab in PROSAIL) = 10:1:39 μg/cm^2^, LAI = 2:0.5:4 m^2^/m^2^, n = 30 × 5=150; (b) Chl-Cab = 21:1:45 μg/cm^2^, LAI = 4.5:0.5:6 m^2^/m^2^, n = 25 × 4=100; (c) Chl-Cab = 26:1:50 μg/cm^2^, LAI = 6.5:0.5:8 m^2^/m^2^, n = 25 × 4=100; total = 350). The other parameters (fixed parameters in Table 3) were determined from a previous study [32]; this was done for ease of implementation in analyzing the responses of VNAI and other Chl VIs to Chl content.

### Generating broadband canopy spectral data from hyperspectral measurements

**Fig. 2:**
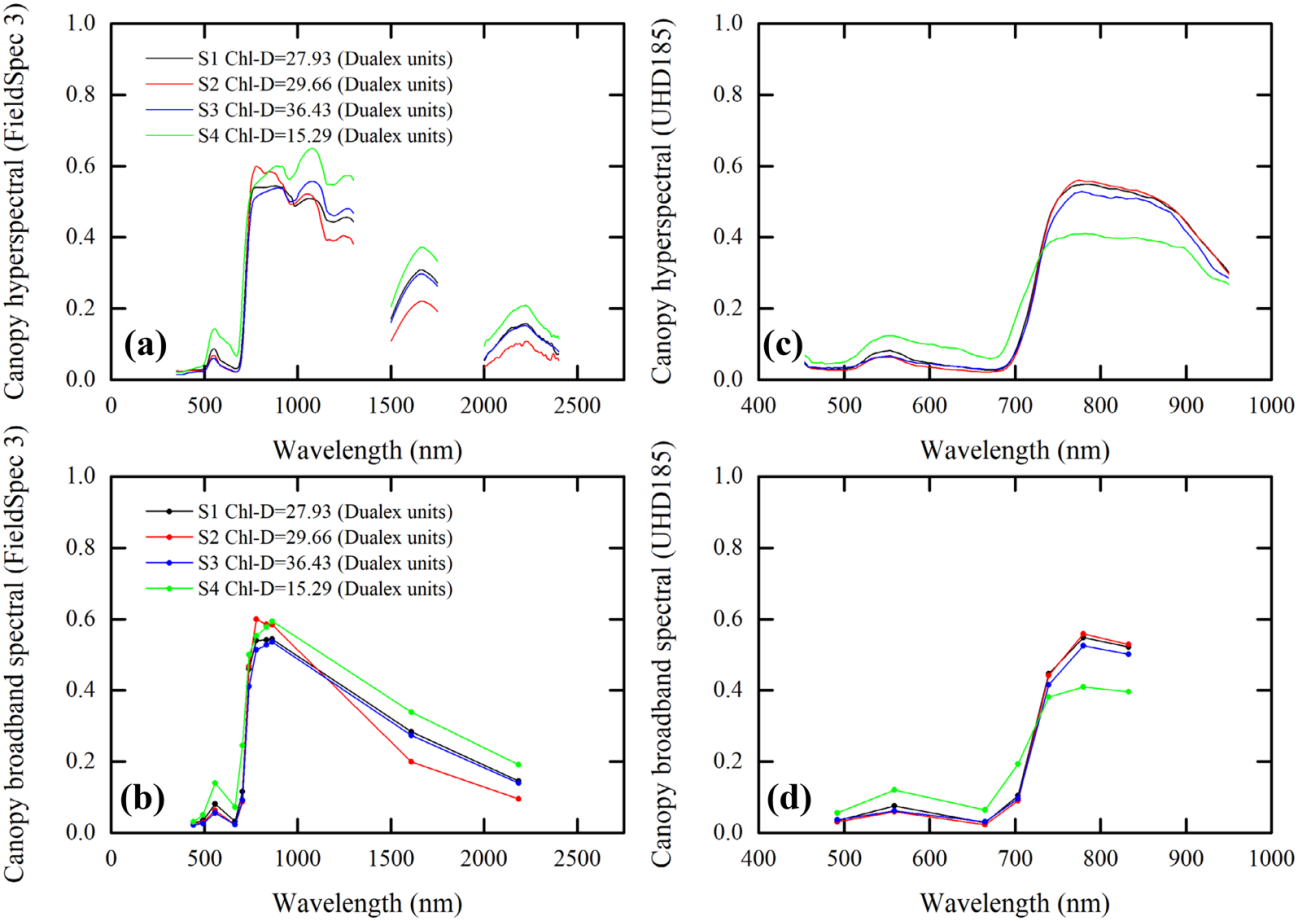
Soybean canopy hyperspectral measurements and corresponding broadband spectral. **a** Field-based soybean canopy hyperspectral measurements and **b** corresponding broadband spectral. **c** Unmanned aerial vehicle (UAV)-based soybean canopy hyperspectral measurements and **d** corresponding broadband spectral

- Traditional satellite-based multispectral sensors can only provide broadband remote sensing spectral data. Thus, using broadband remote sensing spectral data from cost-free satellite-based remote sensing sensors to estimate Chl content has attracted significant attention. The spectral response function (SRF) describes the sensitivity of the photosensor to optical radiation of different wavelengths [66–68]. Usually, the SRF is a.txt file which records the spectral response of the remote sensing sensors to different wavelengths (see Additional file 1: Fig. S1). The data recorded by the multispectral remote sensing sensors is the sum of the products of SRF and surface radiation [67]. Since most available satellite-based remote sensing sensors are multispectral, the hyperspectral reflectance should be converted to multispectral reflectance data to meet the requirements of practical application [66]. Normally, SRF is determined by the optical properties of the multispectral remote sensing sensors. In this work, we used a Sentinel-2 multispectral instrument (MSI) SRF (for the spectral bands for the Sentinel-2 MSI sensors, see Additional file 1: Table S1) to transform the field- and PROSAIL-based hyperspectral data to corresponding broadband remote sensing spectral data. The field soybean canopy MSI multispectral data of each band is the (a) the sum of the products of SRF and hyperspectral reflectance divided by (b) the sum of SRF. Figure 2 shows (i) the field- and UAV-based soybean canopy hyperspectral measurements and (ii) corresponding broadband spectral data calculated from hyperspectral measurements.

## Methods

### Estimation of Chl content based on traditional spectral VIs

Many spectral VIs have been widely used for estimating Chl content. These VIs can be divided into two categories: (i) multiple-bioparameter VIs (e.g., the Normalized Difference Vegetation Index [NDVI], OSAVI, Enhanced Vegetation Index [EVI], Two-band EVI [EVI2]; for references and equations, see Table 4, Type 1) and (ii) pigment VIs (e.g., Pigment-specific Normalized Difference [PSND], Transformed Chlorophyll Absorption Reflectance Index [TCARI]/OSAVI; for references and equations, see Table 4, types 2 and 3). Multiple-bioparameter VIs can be used to extract multiple vegetation parameters, such as LAI, canopy coverage, and pigment content (e.g., Chl). Pigment VIs are suitable for extracting the concentrations of pigments in green vegetation leaves from leaf spectral reflectance, however they are easily affected by canopy and soil background effects. Pigment VIs can be divided into two categories: (i) broadband pigment VIs, which can be calculated using several types of broadband remote sensing data (see Table 4, Type 2); (ii) red-edge-based pigment VIs, which can be calculated using red-edge-band and broadband remote sensing data (see Table 4, Type 3). Table lists several Chl VIs that were used in previous studies. These traditional spectral VIs were established based on the band mathematics. In practice, LAI has the greatest effect on the reflectance in the visible and NIR bands, which reduces the ability of the indices shown in Table 4 to estimate Chl content.

**Table 4:** A summary of some chlorophyll (Chl) vegetation indices (VIs) which were used to estimate the Chl content of vegetation in previous studies

Type	VI	Equation	References
(1)	Normalized Difference VI (NDVI)	$\frac{NIR - R}{NIR + R}$	[69]
	Optimized Soil-adjusted VI (OSAVI)	$\frac{1.16(NIR - R)}{NIR + R + 0.16}$,	[70]
	Enhanced VI (EVI)	$\frac{2.5(NIR - R)}{{NIR{ + 6} \times R - 7.5 \times B + 1}}$	[71]
	Two-band Enhanced VI (EVI2)	$\frac{{2.5\left( {NIR - R} \right)}}{R + 2.4 \times R + 1}$	[72]
(2)	Renormalized Difference VI (RDVI)	$\frac{NIR - R}{{(NIR{ + }R)^{1/2} }}$	[73]
	Pigment-specific Normalized Difference Index (PSND)	$\frac{NIR - B}{NIR + B}$	[20]
	Transformed Chlorophyll Absorption Reflectance Index (TCARI)/OSAVI	$\frac{{ 3 ( ( {\text{NIR}} - {\text{R)}} - 0. 2 ( {\text{NIR - G)(NIR/R))}}}}{{ ( 1 { + 0} . 1 6 ) ( {\text{NIR}} - {\text{R)/(NIR + R + 0}} . 1 6 )}}$	[55]
(3)	Red-edge Chlorophyll Index (CI(red edge))	$\frac{RE3}{RE1} - 1$	[50]
	Normalized Difference Red-edge Version 1 (NDRE1)	$\frac{RE2 - RE1}{RE2 + RE1}$	[54]
	Normalized Difference Red-edge Version 2 (NDRE2)	$\frac{RE3 - RE 1}{RE3 + RE 1}$	[57]
	Red-edge-based Transformed Chlorophyll Absorption Reflectance Index/OSAVI (TCARI/OSAVI_RE)	$\frac{{ 3 ( ( {\text{RE1}} - {\text{R)}} - 0. 2 ( {\text{RE1}} - {\text{G)(RE1/R))}}}}{{ ( 1 { + 0} . 1 6 ) ( {\text{NIR}} - {\text{R)/(NIR + R + 0}} . 1 6 )}}$	[55]

B, G, R, RE1, RE2, RE3, and NIR refer to (i) the blue, green, red, first red-edge, second red-edge, third red-edge, and NIR bands, respectively, of Sentinel-2 MSI (see Additional file 1: Table S1), and (ii) wavelengths of 494, 558, 662, 706, 742, 782, and 830 nm, respectively, in UAV-based UHD 185 images. Types 1, 2, and 3 refer to multiple-bioparameter VIs, broadband pigment VIs, and red-edge-based pigment VIs, respectively

### Proposed Chl index based on the angles between visible and NIR band reflectance

**Fig. 3:**
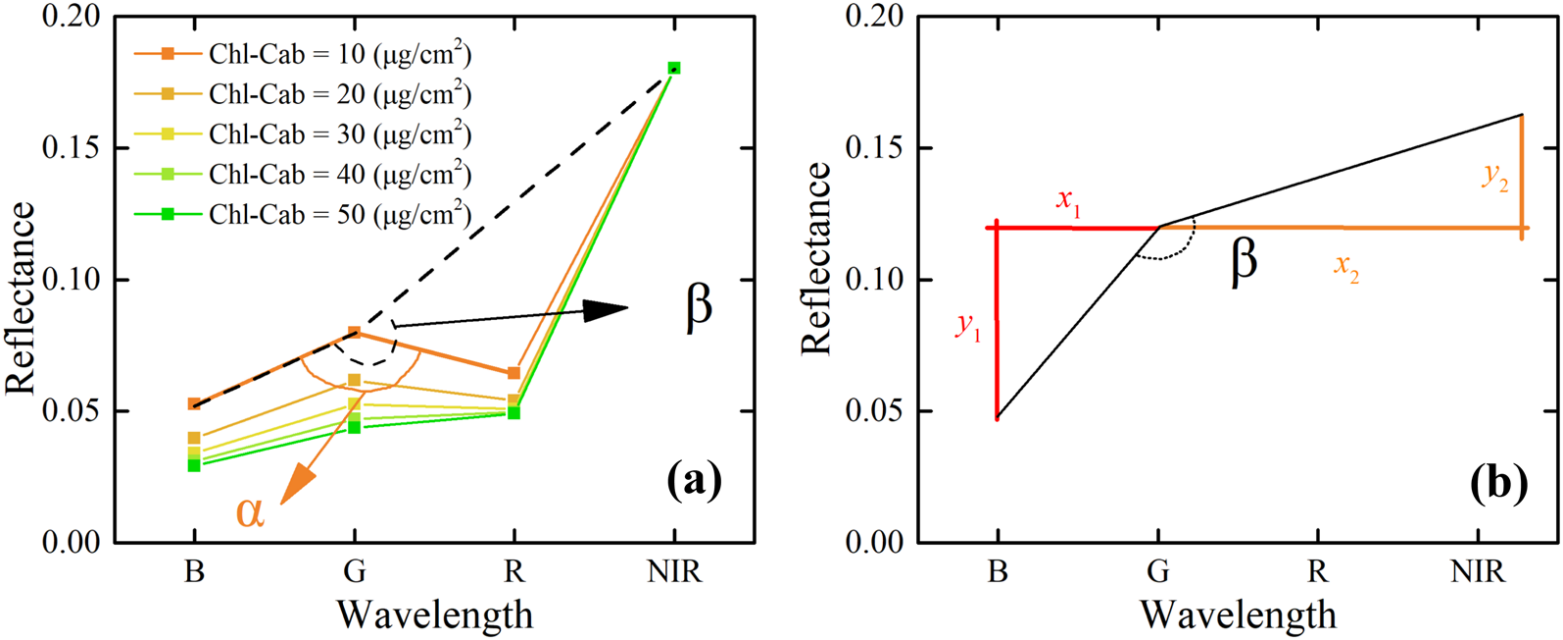
Depictions of the angles α and β which were used to calculate the Visible and NIR Angle Index (VNAI) and their calculation methods. The spectral data in this figure were simulated by using the PROSAIL (a combination of the Properties Optique Spectrales des Feuilles [PROSPECT] model and the Scattering by Arbitrarily Inclined Leaves [SAIL] model) radiative transfer model (RTM) (Leaf Area Index [LAI]: 2 m^2^/m^2^; Chl-Cab (parameter Cab in PROSAIL): 10, 20, 30, 40, 50 μg/cm^2^; see Table for other fixed parameters). The central wavelengths of bands B, G, R, and NIR are 492.4, 559.8, 664.6, and 832.8 nm, respectively

In this study, we proposed and evaluated a VNAI to obtain estimates of soybean canopy Chl content using broadband visible and NIR remote sensing. The VNAI can be explained as the sum of two angles (VNAI = α + β; see Fig. 3). The angle α (Fig. 3a) is the included angle between (i) the line from the reflectance in the blue band to the reflectance in the green band and (ii) the line from the reflectance in the red band to the reflectance in the green band. The angle β (Fig. 3a) is the included angle between (i) the line from the reflectance in the blue band to the reflectance in the green band and (ii) the line from the reflectance in the NIR band to the reflectance in the green band.

Taking the angle β as an example, Fig. 3b shows the calculation method of the three bands-based angle (B, G, and NIR); mathematically, the angle can be calculated as follows:

1 $$\begin{gathered} Angle = 180 - atan\left( {\frac{{y1}}{{x1}}} \right) + atan\left( {\frac{{y2}}{{x2}}} \right), \hfill \\ x1 = wav\left( {G - B} \right),y1 = Ref_{G} - Ref_{B} , \hfill \\ x2 = wav\left( {NIR - G} \right),y1 = Ref_{{NIR}} - Ref_{G} \hfill \\ \end{gathered}$$

where *Ref*_*B*_, *Ref*_*G*_, and *Ref*_*NIR*_ are the spectral reflectance at the central wavelengths of B, G, and NIR, respectively; and wav (*G*-*B*) and wav (*NIR*-*G*) represent the normalized distance of the central wavelengths of (i) bands B and G and (ii) bands G and NIR obtained using;

2 $$\begin{array}{ll} wav\left( {G - B} \right) = \left( {G_{cw} - B_{cw} } \right)/2500 \hfill \\ wav\left( {NIR - G} \right) = \left( {NIR_{cw} - G_{cw} } \right)/2500 \hfill \\ wav\left( {R - G} \right) = \left( {R_{cw} - G_{cw} } \right)/2500 \hfill \\ \end{array}$$

where *B*_cw_, *G*_cw_, *R*_cw_, and *NIR*_cw_ represent the central wavelengths of bands B, G, R, and NIR, which are 492.4, 559.8, 664.6, and 832.8 nm, respectively (see Additional file 1: Table S1); 2500 nm is the maximum wavelength of the optical remote sensing hyperspectral bands. wav (G–B) = (559.8–492.4)/2500 = 0.027, wav(R–G)/2500 = (664.6–559.8)/2500 = 0.0419, and wav(NIR–G)/2500 = (832.8–559.8)/2500 = 0.1092. The angles α and β and the VNAI can be calculated as follows:

3 $$\begin{array}{ll} Angle \alpha = 180 - atan\left( {\frac{{Ref_{G} - Ref_{B} }}{{wav\left( {G - B} \right)}}} \right) + atan\left( {\frac{{Ref_{R} - Ref_{G} }}{{wav\left( {R - G} \right)}}} \right), \hfill \\ Angle \beta = 180 - atan\left( {\frac{{Ref_{G} - Ref_{B} }}{{wav\left( {G - B} \right)}}} \right) + atan\left( {\frac{{Ref_{NIR} - Ref_{G} }}{{wav\left( {NIR - G} \right)}}} \right), \hfill \\ \end{array}$$

4 $$VNAI = \alpha + \beta$$

where *Ref*_*B*_, *Ref*_*G*_, *Ref*_*R*_, and *Ref*_*NIR*_ are the spectral reflectances at the central wavelengths of bands B, G, R, and NIR of the spectral reflectance.

### Datasets, experimental methodology, mapping, and statistical analysis

In this work, three datasets were used to evaluate the estimation performance of Chl content. As shown in Table 5, the PROSAIL-based canopy spectral data were designated as the simulated dataset (obtained using the PROSAIL RTM, n = 350), while the field-based (obtained using the FieldSpec 3 spectrometer, n = 206 + 370 = 576) and UAV-based (obtained using the UHD185 spectrometer, n = 195) canopy spectral data were designated as the real (field) and real (UAV) datasets, respectively.

**Table 5:** Details of the simulated, real (field), and real (UAV) datasets

Dataset	Type	Stage	Number of plots	Calibration	Validation	Total number of data
Simulated	PROSAIL	–	350	–	–	350
Real (field)	Field A	S1	51	–	–	206
		S2	51	–	–	
		S3	51	–	–	
		S4	42	–	–	
		S5	11	–	–	
	Field B	S1	76	–	–	370
		S2	76	–	–	
		S3	76	–	–	
		S4	76	–	–	
		S5	66	–	–	
Real (UAV)	Field A	S1	51	39	12	195
		S2	51	32	19	
		S3	51	36	15	
		S4	42	25	17	
		Total	–	132	63	–

– denotes no data

**Fig. 4:**
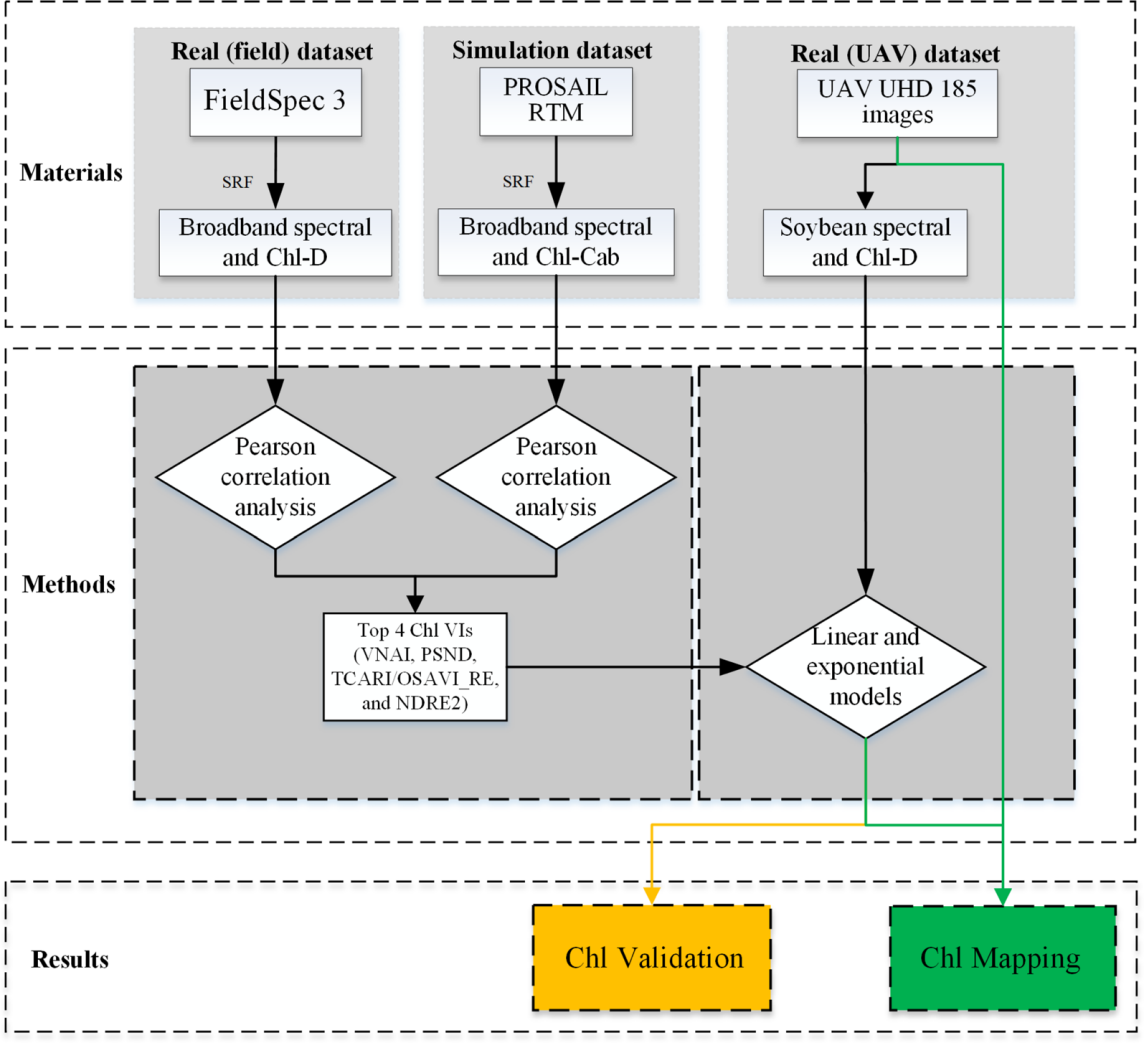
A schematic representation of the experimental methodology. VI: vegetation index. Chl: chlorophyll. SRF: spectral response function. Chl-D: soybean leaf Chl content dataset measured using a Dualex 4 scientific portable sensor. PSND: Pigment-specific Normalized Difference. NDRE2: Normalized Difference Red-edge Version 2. TCARI/OSAVI_RE: Red-edge-based Transformed Chlorophyll Absorption Reflectance Index/Optimized Soil-adjusted Vegetation Index

As shown in Fig. 4, a simulated dataset, a field dataset and a UAV-based dataset were used to evaluate the performance of the VNAI index and 11 traditional Chl VIs. The simulated dataset was used to evaluate the responses of the 11 traditional Chl VIs and the proposed VNAI to Chl-Cab and LAI. Then, the three best-performing Chl VIs were selected for performance comparison using a field dataset. Finally, the Chl estimation performances of these three best-performing Chl VIs and the VNAI were evaluated using UAV-based soybean canopy remote sensing data for growth stages S1–S4, including (i) a calibration dataset consisting of 132 samples and (ii) a validation dataset consisting of 63 samples. Crop canopy parameters (e.g., LAI, fractional vegetation cover [FVC]) differ greatly in different growth stages, and thus linear and exponential regression equations were used to evaluate the performances of the VNAI and other Chl indices for each growth stage for the estimation of soybean Chl.

Additionally, four bands from the UAV-based remote sensing images—namely 494 (blue), 558 (green), 662 (red), and 830 nm (NIR)—and the VNAI were used to map the Chl content of the soybean canopy. Furthermore, the coefficient of determination (*R*^2^), root-mean-square error (RMSE), and mean absolute error (MAE) were used to evaluate the Chl estimation performance of each Chl VI.

## Results

### Response of VNAI to Chl-Cab and LAI

**Fig. 5:**
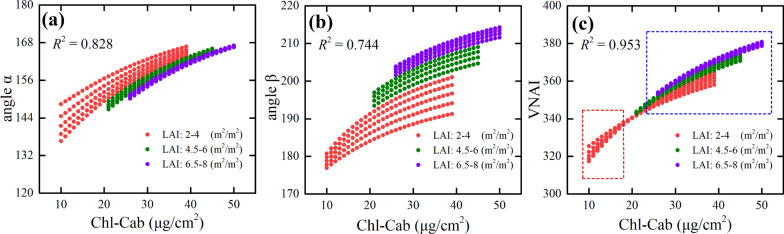
The responses of the angles α (**a**) and β (**b**), and the VNAI (**c**) to Chl-Cab and LAI (LAI: 2:0.5:8 m^2^/m^2^). Red and blue boxes represent low (Chl-Cab < 20 μg/cm^2^) and high (Chl-Cab > 20 μg/cm^2^) Chl-Cab samples

Figure 5 shows the responses of the angles α and β, and the VNAI, to Chl-Cab and LAI. As shown in Fig. 5a and b, the angles α (*R*^2^ = 0.828) and β (*R*^2^ = 0.744) of the vegetation canopy spectral reflectance increase as Chl-Cab increases.

However, the effect of the LAI on α and β may reduce their sensitivity to Chl-Cab. As the LAI increases, the angle α decreases and the angle β increases (Fig. 5). The effects of the LAI on α and β were mitigated by using the sum of α and β. As shown in Fig., the correlation between Chl-Cab and the VNAI (*R*^2^ = 0.953) is greater than the correlation between Chl-Cab and α (*R*^2^ = 0.828) and β (*R*^2^ = 0.744).

### Response of PROSAIL-based Chl VIs to Chl-Cab and LAI

**Fig. 6:**
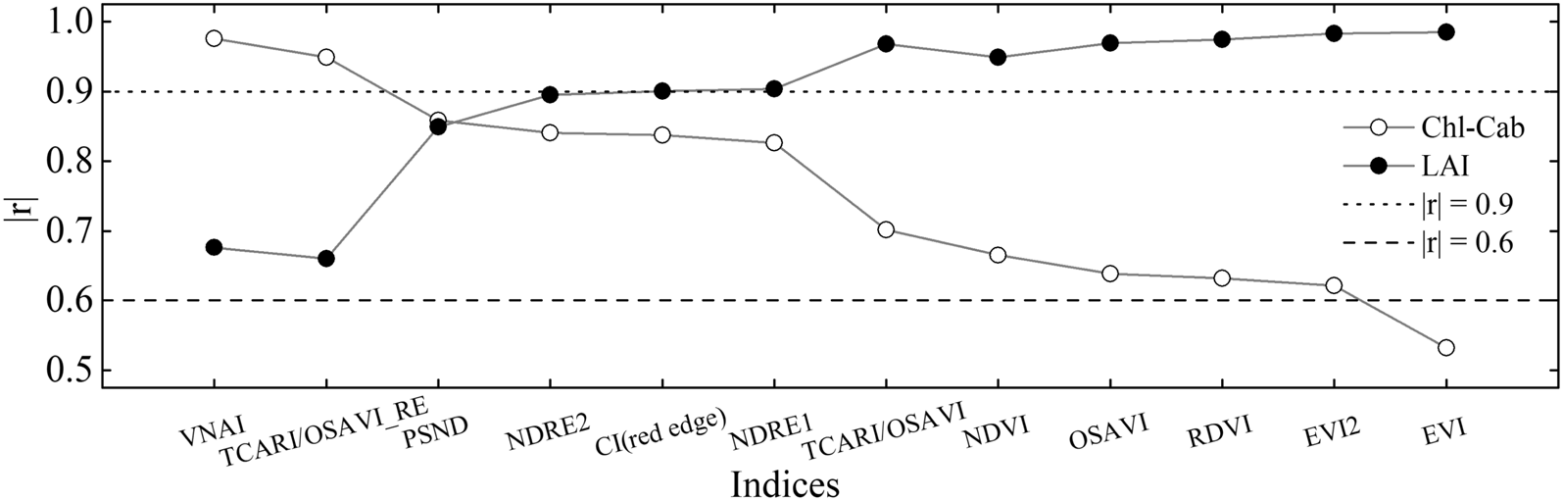
The absolute values of the Pearson correlation coefficient (|*r*|) between (i) Chl VIs and (ii) Chl-Cab and LAI (simulated dataset, n = 350). Note: OSAVI: Optimized Soil-adjusted VI. TCARI/OSAVI_RE: Red-edge-based Transformed Chlorophyll Absorption Reflectance Index/OSAVI. PSND: Pigment-specific Normalized Difference Index. NDRE2: Normalized Difference Red-edge Version 2. CI(red edge): Red-edge Chlorophyll Index. NDRE1: Normalized Difference Red-edge Version 1. TCARI/OSAVI: Transformed Chlorophyll Absorption Reflectance Index/OSAVI. NDVI: Normalized Difference Vegetation Index. RDVI: Renormalized Difference Vegetation Index. EVI: Enhanced Vegetation Index. EVI2: Two-band EVI

Figure 6 presents the Pearson correlation coefficients between the Chl VIs and (i) Chl-Cab and (ii) the LAI, for the simulated dataset. The results show that the Chl VIs were correlated with Chl-Cab and LAI to varying degrees. For Chl-Cab, the results indicate that the correlation coefficients increased in the following order: VNAI > TCARI/OSAVI_RE > PSND > NDRE2 > CI(red edge) > NDRE1 > TCARI/OSAVI > NDVI > OSAVI > RDVI > EVI2 > EVI. Furthermore, in general, VIs which were highly correlated with LAI were weakly correlated with Chl-Cab (Fig. 6). The use of traditional Chl VIs to estimate canopy Chl content may be hindered by variations in the crop LAI. Based on the results of the Pearson correlation analysis shown in Fig. 6, the three best-performing existing Chl VIs (TCARI/OSAVI_RE, PSND, and NDRE2) were selected for comparison with the proposed VNAI.

**Fig. 7:**
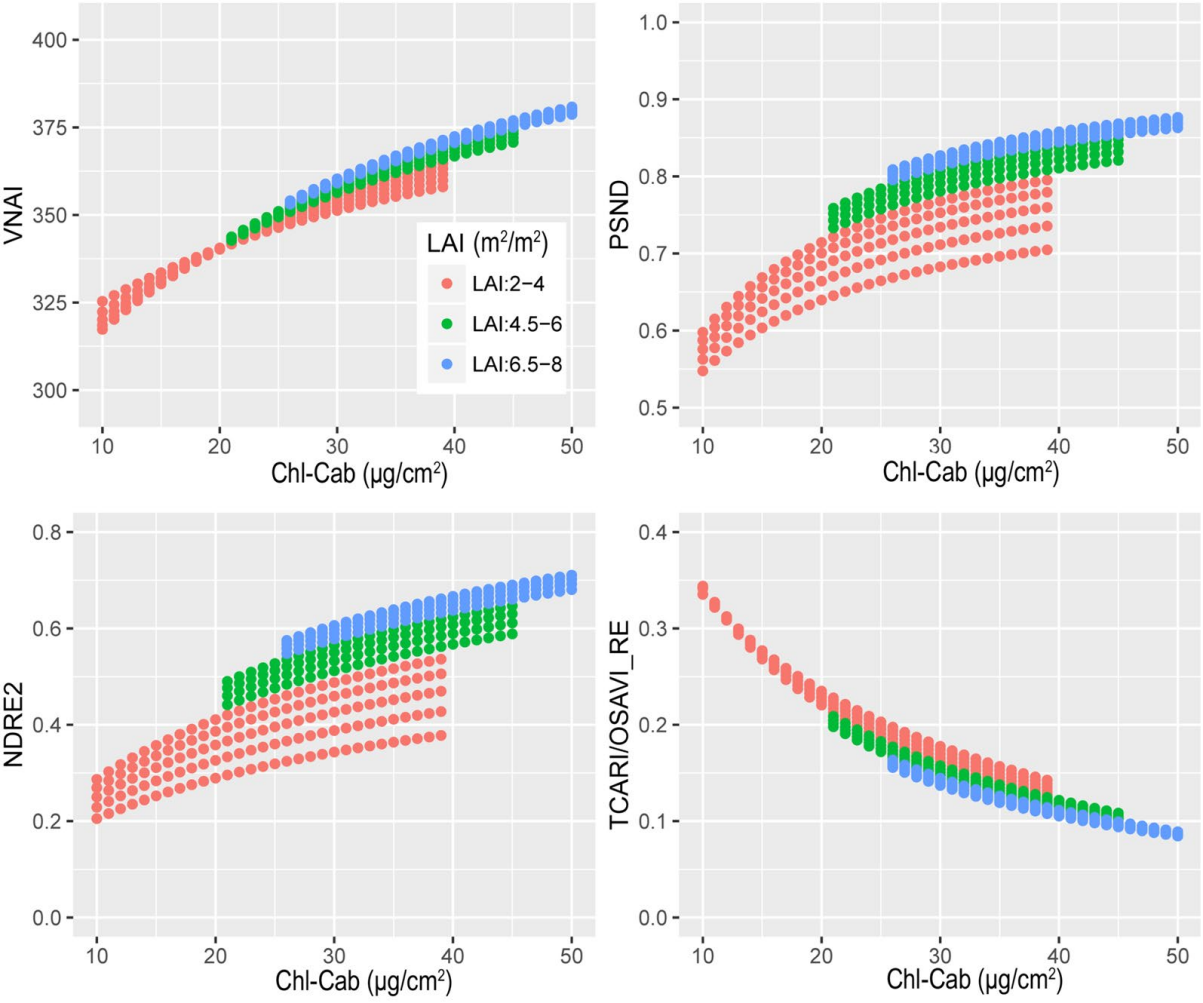
The responses of the VNAI, PSND, NDRE2, and TCARI/OSAVI_RE to Chl-Cab (μg/cm^2^) and LAI (m^2^/m^2^) (simulated dataset, n = 350)

Figure 7 shows the four VIs (VNAI, TCARI/OSAVI_RE, PSND, and NDRE2) as a function of Chl-Cab and LAI using the simulated dataset (see Table 5). With increasing LAI, large changes were observed in PSND and NDRE2; However, with increasing LAI, small changes in the VNAI were observed (Fig. 7). Taking PSND as an example, (i) the PSND exhibited a logarithmic relationship with Chl-Cab and (ii) samples with a high LAI showed a higher PSND than samples with a low LAI (Fig. 7). This indicates that the LAI may reduce the PSND-based Chl estimation performance. The results in Fig. 7 indicate that the canopy effect (LAI) has a significant influence on the Chl VIs.

#### Response of field-based Chl VIs to Chl-D

**Fig. 8:**
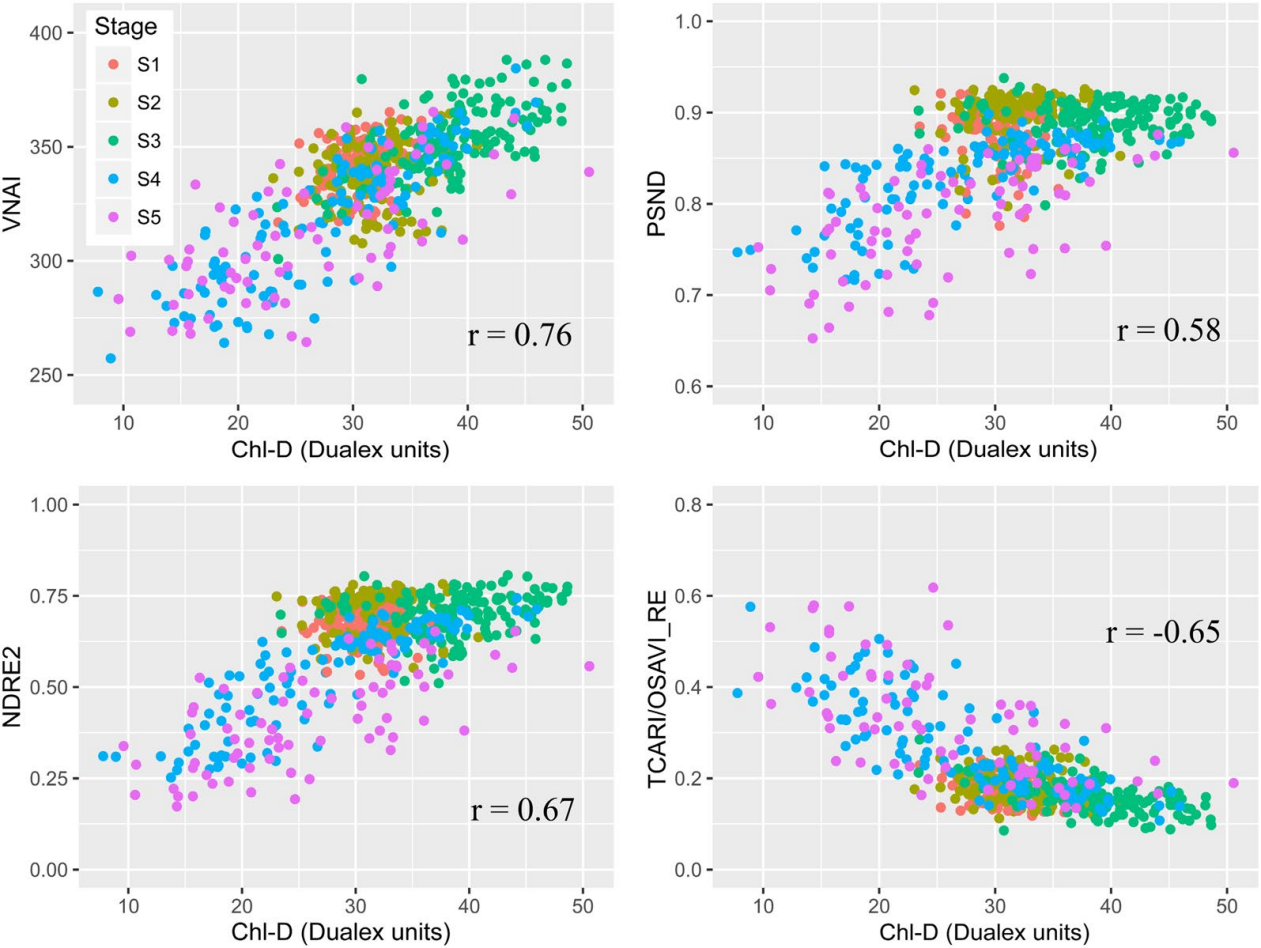
Responses of VNAI, PSND, NDRE2, and TCARI/OSAVI_RE to Chl-D (real (field) dataset, n = 576)

Figure 8 shows the four VIs as a function of Chl-D by using the real (field) dataset (see Table 5). The results indicate that the proposed VNAI has the highest Pearson correlation coefficient of the four selected VIs, which is similar to the results for the simulated dataset shown in Fig. 7. Additionally, the results indicate that PSND and NDRE2 were saturated when Chl-D = 30 (Fig. 8), which is similar to the findings for the simulated dataset (Fig. 7).

### Response of UAV-based Chl VIs to Chl-D

**Fig. 9:**
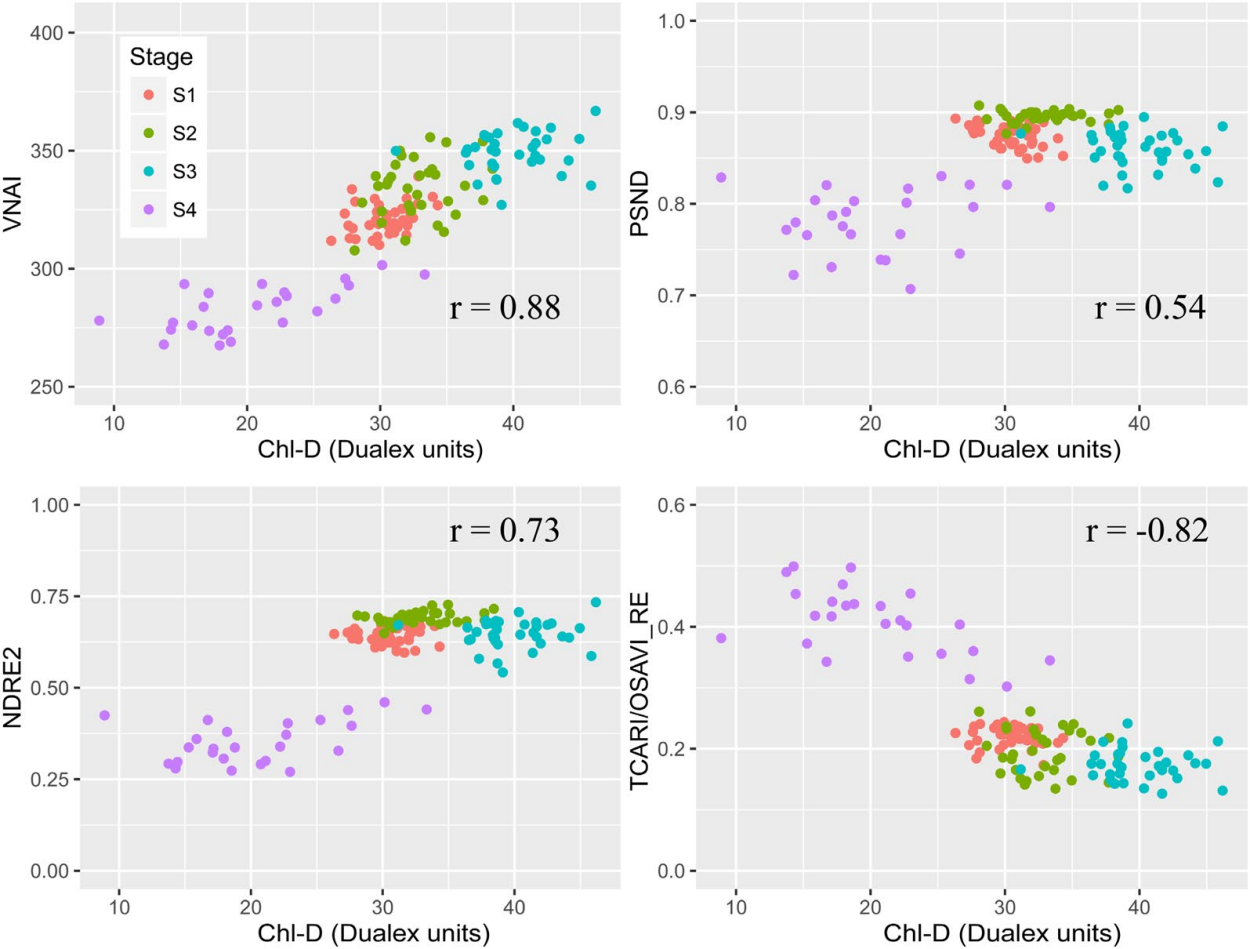
The responses of real (UAV) Chl VIs to Chl-D (n = 132)

Figure 9 shows the four Chl VIs as a function of Chl-D for the real (UAV) calibration dataset (see Table 5). The relationship between the four VIs and Chl-D for Field A was similar to that of the real (field) dataset (Fig. 9). The relationship between Chl-D and the Chl VIs is shown in Table 6. The Chl-D estimated with PSND and NDRE2 all affected by canopy effects, with *R*^2^ ranging from 0.27 to 0.53, MAE ranging from 4.18 to 4.98, and RMSE ranging from 5.03 to 6.37. The Chl-D estimation accuracy using TCARI/OSAVI_RE was lower than that of the VNAI, with an *R*^2^ of 0.67, MAE ranging from 3.28 to 3.33, and RMSE ranging from 4.21 to 4.23. The linear-based Chl-D estimates using the proposed VNAI (Table 6) demonstrated promising results, with an *R*^2^ of 0.77, an MAE of 2.73, and an RMSE of 3.54.

**Table 6:** The relationships between the UAV-based Chl VIs and Chl-D (Dualex units) shown in Fig

VI	Equation	*R*^2^	MAE	RMSE
VNAI	(e1) *Chl*-*D* = 0.2622 × *VNAI* – 53.473	0.77	2.73	3.54
	(e2) *Chl*-*D* = 1.3074 × EXP (0.0097 × *VNAI*)	0.74	3.00	3.79
PSND	(e3) *Chl*-*D* = 90.91 × *PSND *– 46.337	0.29	4.91	6.18
	(e4) *Chl*-*D* = 1.212 × EXP (3.763 × *PSND*)	0.27	4.98	6.37
TCARI/OSAVI_RE	(e5) *Chl*-*D* = –66.358 × *TCARI/OSAVI_RE *+ 47.353	0.67	3.33	4.21
	(e6) *Chl*-*D* = 56.11 × EXP (–2.561 × *TCARI/OSAVI_RE*)	0.67	3.28	4.23
NDRE2	(e7) *Chl*-*D* = 42.353 × *NDRE2* + 6.2227	0.53	4.18	5.03
	(e8) *Chl*-*D* = 11.158 × EXP (1.6807 × *NDRE2*)	0.51	4.22	5.17

Equations e1, e3, e5, and e7 were used for the validation of the Chl models. *R*^2^ coefficient of determination, *MAE* mean absolute error, *RMSE* root-mean-square error

### Chl mapping

**Fig. 10:**
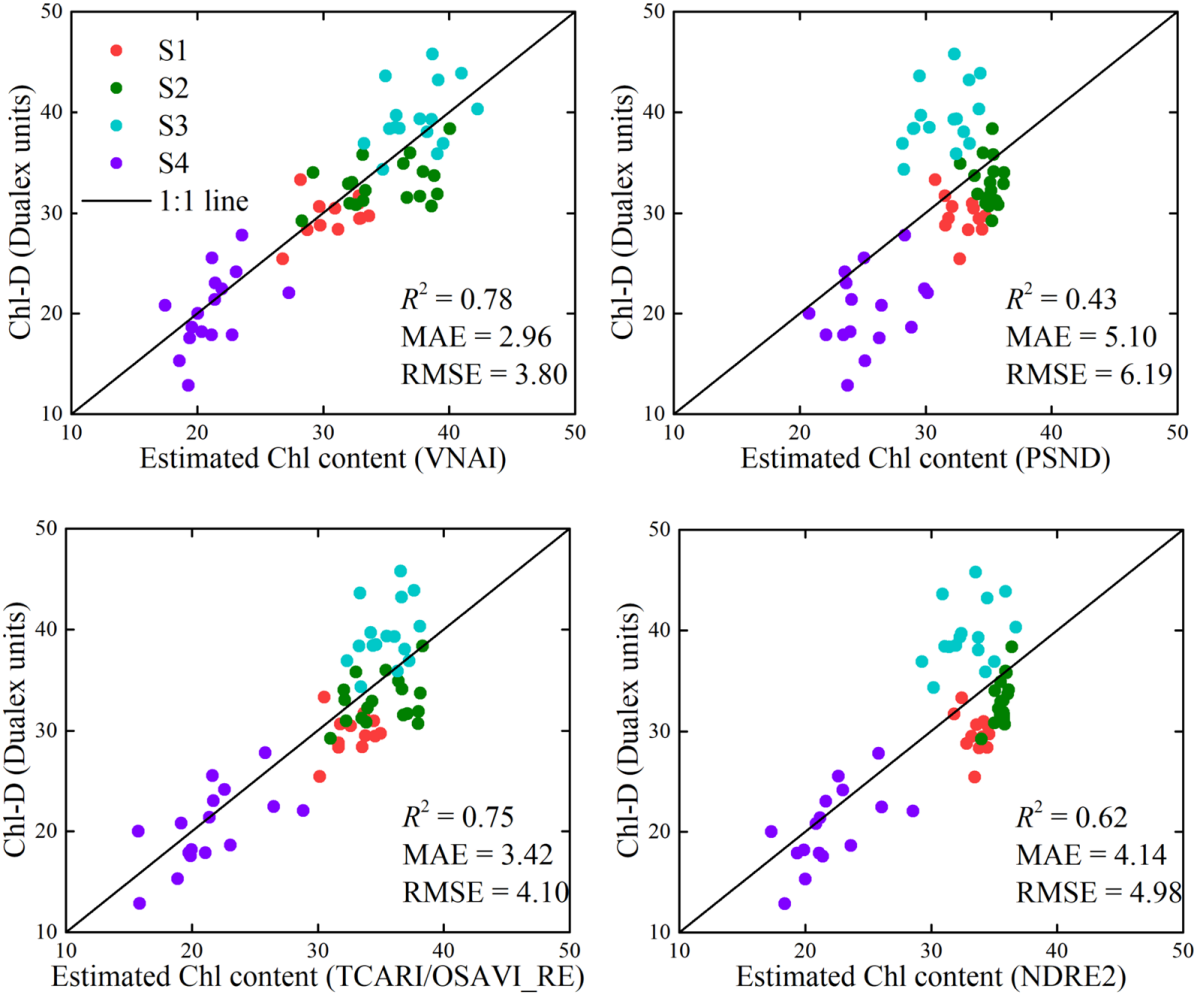
Measured and estimated Chl contents using the real (UAV) validation dataset (n = 63). Note: MAE: mean absolute error; RMSE: root-mean-square error

**Fig. 11:**
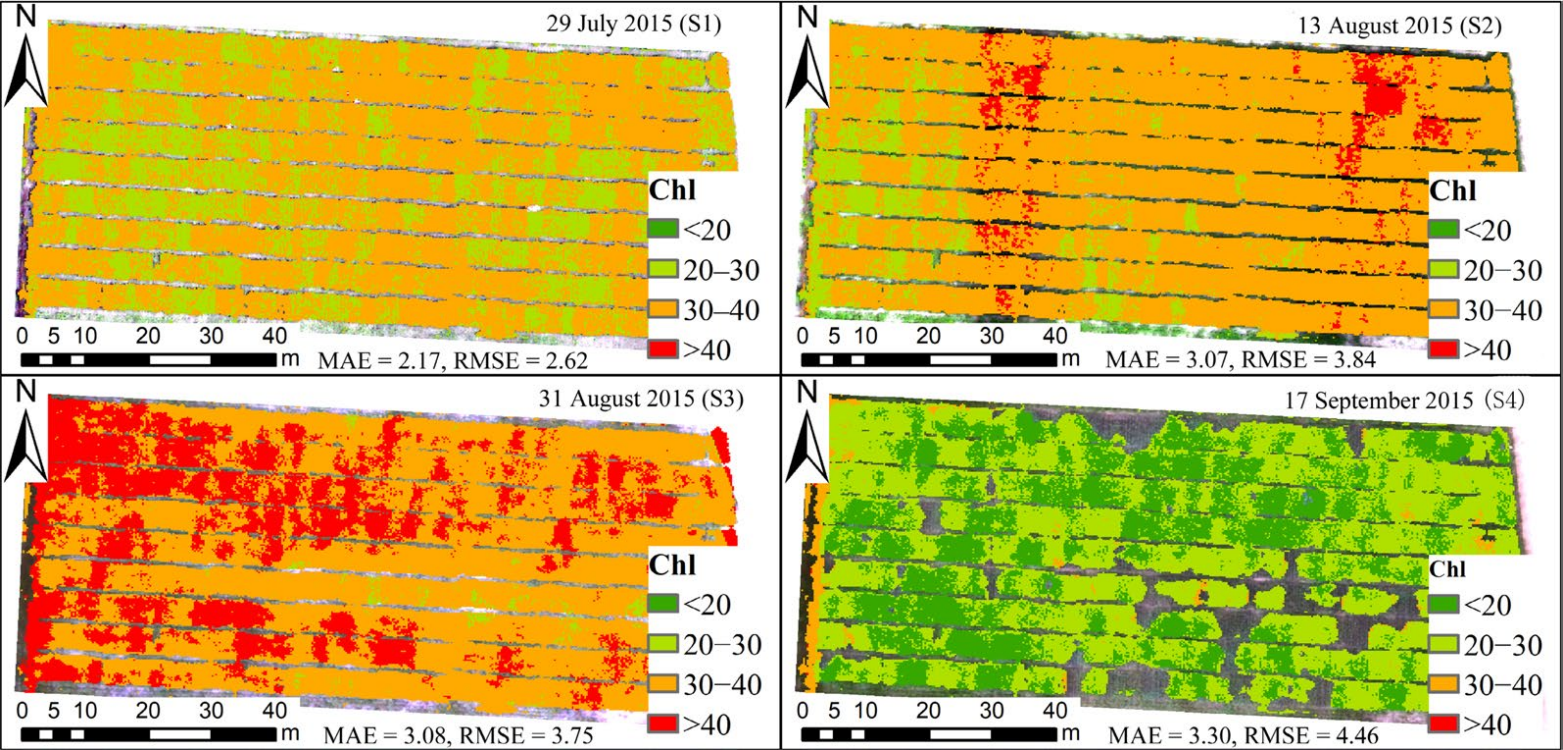
Maps of Chl content (Dualex units) for the four growth stages (S1–S4, NDVI > 0.3 pixels). Note: MAE(S1) = 2.17, RMSE(S1) = 2.62; MAE(S2) = 3.07, RMSE(S2) = 3.84; MAE(S3) = 3.08, RMSE(S3) = 3.75; MAE(S4) = 3.30, RMSE(S4) = 4.46

We used (i) the VNAI and e1, (ii) PSND and e3, (iii) TCARI/OSAVI_RE and e5, and (iv) NDRE2 and e7 to estimate the Chl content (e1, e3, e5, and e7, see Table 6). The estimated and measured values of Chl content are shown in Fig. 10. The results indicate that the VNAI performed better than PSND, TCARI/OSAVI_RE, and NDRE2. For example, when using the PSND, the higher Chl-Ds are underestimated (see Fig. 10) whereas the lower Chl-Ds are overestimated (Fig. 10). Furthermore, we mapped the soybean canopy Chl content for the four growth stages (Fig. 11) using the VNAI, the linear equations in Table 6 (e1), and UAV-based remote sensing images. The spatial distribution of the estimated Chl content in the four stages indicates that the canopy Chl first increased and then decreased with soybean growth (stages S1–S4), which is consistent with the field measurements of Chl content (Table 2, Fig. 10).

## Analysis and discussion

### Chl content estimation performance of VNAI versus (a) existing broadband Chl VIs and (b) red-edge Chl VIs

Existing broadband Chl VIs primarily depend on simple band mathematics, and can be divided into the following three categories: (i) simple ratio VIs, (ii) normalized difference VIs (e.g., NDVI [69] and PSND [20]), and (iii) modified VIs (e.g., OSAVI [70]). The accuracy of Chl estimates based on existing broadband Chl VIs was determined by using the optimal band combination. Almost all two-band-based combinations have been tested in previous studies; However, the band-mathematics-based broadband Chl VIs obtained a low correlation with the simulated dataset (Fig. 6).

Since this study focuses on the estimation of Chl content using visible and NIR broadband remote sensing data, all of the selected broadband VIs (NDVI, OSAVI, EVI, EVI2, RDVI, PSND, and TCARI/OSAVI; see Table 4 and Fig. 6) were calculated using visible and NIR broadband remote sensing spectral data. The results shown in Fig. 6 indicate that all previously used broadband Chl VIs demonstrated a significant correlation with LAI, thereby indicating that LAI has a significant influence on previous band-mathematics-based (i) multiple-bioparameter VIs (NDVI, OSAVI, EVI, EVI2; see Pearson correlation coefficients in Fig. 6) and (ii) broadband pigment VIs (PSND, TCARI/OSAVI; see Pearson correlation coefficients in Fig. 6). This phenomenon contributed to the performance in the multi-stage estimation of Chl content, which was consistent with previous research results [41, 42]. In contrast, the proposed broadband VNAI was shown to be more sensitive to Chl content than to the LAI, thus indicating that the VNAI may have a more robust relationship with Chl content (Fig. 6).

Aside from the VNAI, some other promising red-edge-based Chl VIs were investigated in this paper (CI(red edge), NDRE1, NDRE2, and TCARI/OSAVI_RE; see Table and Fig. 6). The results shown in Fig. 6 indicate that the VNAI-based estimates of Chl content were superior to those based on NDRE1, NDRE2, and CI(red edge). The performance of the broadband VNAI and the red-edge-based TCARI/OSAVI_RE were found to be similar when using the simulated dataset (Fig. 6); however, the VNAI obtained more accurate estimates of Chl content when using the real datasets (Fig. 8, real (field) dataset: r (VNAI) = 0.76, r (TCARI/OSAVI_RE) = -0.65; Fig. 9, real (UAV) dataset: r (VNAI) = 0.88, r (TCARI/OSAVI_RE) = -0.82). This may be due to the fact that (i) the soybean LAI varies tremendously during multi-stage field measurement and (ii) the canopy spectral angles (α + β) were less affected by LAI than by the 11 other selected indices.

The responses of the selected Chl VIs to Chl content and LAI are different. The results of this study show the following: (a) Traditional multiple-bioparameter VIs and broadband pigment VIs are sensitive to LAI (Fig. 6); thus, traditional multiple-bioparameter VIs and broadband pigment VIs may not suitable for estimating crop Chl content over multiple growth stages or in locations where LAI varies greatly; (b) The Pearson correlation coefficients between Chl VIs and (i) Chl-Cab and (ii) LAI indicate that the performances of the VNAI and the red-edge TCARI/OSAVI_RE (the best-performing red-edge VI) are very close (Fig. 6); thus, the VNAI can be used to obtain high-precision Chl maps (Table 6, Figs. 10, 11). Thus, the VNAI can help to obtain accurate estimates of Chl content when using various types of traditional free-access multispectral remote sensing images (e.g., Landsat TM/ETM+/OLI).

### Future applications and limitations of broadband VNAI

In previous studies, other hyperspectral techniques showed promising results for the estimation of the Chl content of vegetation (e.g., band-depth analysis techniques [46, 47], continuous wavelet transform techniques [48, 49]). However, the use of satellite hyperspectral images increases the computation and storage burden. Furthermore, narrowband Chl VIs cannot be applied to data from traditional broadband multispectral remote sensing sensors. Additionally, satellite hyperspectral sensors are expensive, scarce, and cannot provide spatial distributions of crop Chl content over large areas with both high temporal resolution and high spatial resolution.

VNAI has more advantages than the wieldy used broadband Chl indices and red-edge Chl indices. Firstly, a remote sensing sensor with four broad bands (red, green, blue, and NIR) is sufficient to accurately estimate the crop canopy Chl content using VNAI, which makes this method cheaper than methods that require remote sensing sensors with red-edge bands, which are very expensive. Additionally, VNAI can be applied to large amounts of satellite-based broadband remote sensing images; for example, the Landsat TM, ETM+, and OLI sensors can obtain broadband remote sensing images with red, green, blue, and NIR bands. In this case, the VNAI can also be applied to satellite remote sensing images for estimating large-scale vegetation canopy Chl content.

However, as with any method, VNAI has shortcomings. The calculation method is the most obvious one. The calculation method for VNAI (Eqs. 3 and 4) is relatively complicated compared to that of band-operation-based Chl VIs (Table 4), which may limit the application of VNAI. Additionally, this work only focused on the effects of the canopy on remote sensing-based Chl estimation, and did not consider the effects of soil background. Furthermore, this work analyzed and validated VNAI only for soybean canopy; therefore, in the future, additional quantitative field validation should be conducted to investigate the feasibility of conducting long-term canopy Chl estimation for different crops (e.g., wheat, maize, corn), canopy Chl ranges, and soil background using VNAI and remote sensing images.

## Conclusion

In this work, we developed a broadband VNAI for the estimation of crop canopy Chl content. We evaluated (i) the response of the proposed broadband VNAI and several existing broadband and red-edge Chl VIs to Chl content and LAI, (ii) the performance of Chl content estimation using real (field) soybean canopy spectral data, and (iii) soybean canopy Chl mapping using real (UAV) soybean canopy remote sensing images. The following conclusions could be drawn from this work:

(i) Most previously used broadband Chl VIs were significantly correlated with LAI, and the proposed broadband VNAI was more sensitive to Chl content than to LAI, thereby indicating that the VNAI may have a more robust relationship with Chl content (Figs. 6 and 7).
(ii) With increasing LAI, the angle α decreases and the angle β increases (Fig. 5). The LAI effects were mitigated by using the sum of the angles α and β (Fig. 5).
(iii) The estimates of Chl content based on the broadband VNAI were more accurate than the estimates based on the other investigated VIs (simulated dataset: Fig. 7; real (field) dataset: Fig. 8; real (UAV) dataset: Fig. 9, Table 6).

## Supplementary information

**Additional file 1.** Additional table and figures.

## Data Availability

The datasets used and/or analyzed during the current study are available from the corresponding author on request.

## Abbreviations

Chl: Chlorophyll
CI (red edge): Red-edge Chlorophyll index
COV: Coefficient of variation
DOM: Digital orthophoto map
ETM+: Enhanced Thematic Mapper Plus
EVI: Enhanced VI
EVI2: Two-band Enhanced VI
FVC: Fractional vegetation cover
INFORM: Invertible Forest Reflectance Model
LAI: Leaf area index
LUT: Lookup table
MAE: Mean absolute error
MSI: Multispectral instrument
NDRE: Normalized Difference Red-edge Index
NDRE1: Normalized Difference Red-edge Version 1
NDRE2: Normalized Difference Red-edge Version 2
NDVI: Normalized Difference VI
NIR: Near-infrared
OLI: Operational Land Imager
OSAVI: Optimized Soil-adjusted Vegetation Index
PLSR: Partial least square regression
POS: Position and orientation system
PROSAIL: PROSPECT and SAIL radiative transfer models
PROSPECT: Properties Optique Spectrales des Feuilles
PSND: Pigment-specific Normalized Difference
*R*^2^: Coefficient of determination
RDVI: Renormalized Difference VI
RMSE: Root-mean-square error
ROI: Region of interest
RTM: Radiative transfer model
SAIL: Scattering by Arbitrarily Inclined Leaves
SD: Standard deviation
SRF: Spectral response function
SVR: Support vector machine regression
SWIR: Short-wave infrared
TCARI: Transformed Chlorophyll Absorption Reflectance Index
TCARI/OSAVI: Transformed Chlorophyll Absorption in Reflectance Index/OSAVI
TCARI/OSAVI_RE: Red-edge-based Transformed Chlorophyll Absorption in Reflectance Index/OSAVI
TM: Landsat Thematic Mapper
UAV: Unmanned aerial vehicle
VI: Vegetation index
VNAI: Visible and Near-infrared Angle Index

## References

[1] Houborg R, Cescatti A, Migliavacca M, Kustas WP (2013). Satellite retrievals of leaf chlorophyll and photosynthetic capacity for improved modeling of GPP. Agric For Meteorol.

[2] Ač A, Malenovský Z, Olejníčková J, Gallé A, Rascher U, Mohammed G (2015). Meta-analysis assessing potential of steady-state chlorophyll fluorescence for remote sensing detection of plant water, temperature and nitrogen stress. Remote Sens Environ.

[3] Gorbe E, Calatayud A (2012). Applications of chlorophyll fluorescence imaging technique in horticultural research: a review. Sci Hortic (Amsterdam)..

[4] Meroni M, Rossini M, Guanter L, Alonso L, Rascher U, Colombo R (2009). Remote sensing of solar-induced chlorophyll fluorescence: review of methods and applications. Remote Sens Environ.

[5] Li Z, Wang J, He P, Zhang Y, Liu H, Chang H (2015). Modelling of crop chlorophyll content based on Dualex. Trans Chin Soc Agric Eng..

[6] Sid’ko AF, Botvich IY, Pisman TI, Shevyrnogov AP (2017). Estimation of chlorophyll content and yield of wheat crops from reflectance spectra obtained by ground-based remote measurements. Field Crop Res..

[7] Houborg R, McCabe MF, Cescatti A, Gitelson AA (2015). Leaf chlorophyll constraint on model simulated gross primary productivity in agricultural systems. Int J Appl Earth Obs Geoinf.

[8] Gitelson AA, Peng Y, Arkebauer TJ, Schepers J (2014). Relationships between gross primary production, green LAI, and canopy chlorophyll content in maize: implications for remote sensing of primary production. Remote Sens Environ.

[9] Ramírez DA, Yactayo W, Gutiérrez R, Mares V, De Mendiburu F, Posadas A (2014). Chlorophyll concentration in leaves is an indicator of potato tuber yield in water-shortage conditions. Sci Hortic (Amsterdam)..

[10] Yu K, Lenz-Wiedemann V, Chen X, Bareth G (2014). Estimating leaf chlorophyll of barley at different growth stages using spectral indices to reduce soil background and canopy structure effects. ISPRS J Photogramm Remote Sens..

[11] Pu R, Gong P. Hyperspectral Remote Sensing of Vegetation Bioparameters. In: Advances in Environmental Remote Sensing Sensors, Algorithms, and Applications. CRC Press. 2011. ISBN: 9780429143687. 10.1201/b10599.

[12] Liang S, Li X, Wang J. Advanced Remote Sensing. Academic Press; 2012. ISBN: 978-0-12-815826-5. https://doi.org/10.1016/C2017-0-03489-4

[13] Jacquemoud S (1993). Inversion of the PROSPECT + SAIL canopy reflectance model from AVIRIS equivalent spectra: theoretical study. Remote Sens Environ.

[14] Feret JB, François C, Asner GP, Gitelson AA, Martin RE, Bidel LPR (2008). PROSPECT-4 and 5: advances in the leaf optical properties model separating photosynthetic pigments. Remote Sens Environ.

[15] Verrelst J, Rivera JP, Moreno J. “ARTMO’s global sensitivity analysis (GSA) toolbox to quantify driving variables of leaf and canopy radiative transfer models.” EARSeL eProceedings, Speical 2 (2015): 1–11. 10.12760/02-2015-2-01.

[16] Broge NH, Leblanc E (2001). Comparing prediction power and stability of broadband and hyperspectral vegetation indices for estimation of green leaf area index and canopy chlorophyll density. Remote Sens Environ.

[17] Sun J, Shi S, Yang J, Chen B, Gong W, Du L (2018). Estimating leaf chlorophyll status using hyperspectral lidar measurements by PROSPECT model inversion. Remote Sens Environ.

[18] Houborg R, McCabe M, Cescatti A, Gao F, Schull M, Gitelson A (2015). Joint leaf chlorophyll content and leaf area index retrieval from Landsat data using a regularized model inversion system (REGFLEC). Remote Sens Environ.

[19] Lunagaria MM, Patel HR (2019). Evaluation of PROSAIL inversion for retrieval of chlorophyll, leaf dry matter, leaf angle, and leaf area index of wheat using spectrodirectional measurements. Int J Remote Sens.

[20] Blackburn GA (1998). Quantifying chlorophylls and carotenoids at leaf and canopy scales: an evaluation of some hyperspectral approaches. Remote Sens Environ.

[21] Zarco-Tejada PJ, Berjón A, López-Lozano R, Miller JR, Martín P, Cachorro V (2005). Assessing vineyard condition with hyperspectral indices: leaf and canopy reflectance simulation in a row-structured discontinuous canopy. Remote Sens Environ.

[22] Peñuelas J, Gamon JA, Fredeen AL, Merino J, Field CB (1994). Reflectance indices associated with physiological changes in nitrogen- and water-limited sunflower leaves. Remote Sens Environ.

[23] Atzberger C, Guérif M, Baret F, Werner W (2010). Comparative analysis of three chemometric techniques for the spectroradiometric assessment of canopy chlorophyll content in winter wheat. Comput Electron Agric..

[24] Yue J, Feng H, Yang G, Li Z (2018). A comparison of regression techniques for estimation of above-ground winter wheat biomass using near-surface spectroscopy. Remote Sens..

[25] Darvishzadeh R, Skidmore A, Abdullah H, Cherenet E, Ali A, Wang T (2019). Mapping leaf chlorophyll content from Sentinel-2 and RapidEye data in spruce stands using the invertible forest reflectance model. Int J Appl Earth Obs Geoinf.

[26] Yin C, He B, Quan X, Liao Z (2016). Chlorophyll content estimation in arid grasslands from Landsat-8 OLI data. Int J Remote Sens.

[27] Darvishzadeh R, Matkan AA, Dashti Ahangar A (2012). Inversion of a radiative transfer model for estimation of rice canopy chlorophyll content using a lookup-table approach. IEEE J Sel Top Appl Earth Obs Remote Sens..

[28] Liang L, Qin Z, Zhao S, Di L, Zhang C, Deng M (2016). Estimating crop chlorophyll content with hyperspectral vegetation indices and the hybrid inversion method. Int J Remote Sens.

[29] Singhal G, Bansod B, Mathew L, Goswami J, Choudhury BU, Raju PLN (2019). Chlorophyll estimation using multi-spectral unmanned aerial system based on machine learning techniques. Remote Sens Appl Soc Environ..

[30] Kira O, Linker R, Gitelson A (2015). Non-destructive estimation of foliar chlorophyll and carotenoid contents: focus on informative spectral bands. Int J Appl Earth Obs Geoinf.

[31] Panigada C, Rossini M, Busetto L, Meroni M, Fava F, Colombo R (2010). Chlorophyll concentration mapping with MIVIS data to assess crown discoloration in the Ticino park oak forest. Int J Remote Sens.

[32] Berger K, Atzberger C, Danner M, D’Urso G, Mauser W, Vuolo F (2018). Evaluation of the PROSAIL model capabilities for future hyperspectral model environments: a review study. Remote Sens..

[33] Jacquemoud S, Verhoef W, Baret F, Bacour C, Zarco-Tejada PJ, Asner GP (2009). PROSPECT + SAIL models: a review of use for vegetation characterization. Remote Sens Environ.

[34] Jacquemoud S, Baret F, Andrieu B, Danson FM, Jaggard K (1995). Extraction of vegetation biophysical parameters by inversion of the PROSPECT + SAIL models on sugar beet canopy reflectance data Application to TM and AVIRIS sensors. Remote Sens Environ.

[35] Féret JB, Gitelson AA, Noble SD, Jacquemoud S (2017). PROSPECT-D: towards modeling leaf optical properties through a complete lifecycle. Remote Sens Environ.

[36] Delloye C, Weiss M, Defourny P (2018). Retrieval of the canopy chlorophyll content from Sentinel-2 spectral bands to estimate nitrogen uptake in intensive winter wheat cropping systems. Remote Sens Environ.

[37] Golhani K, Balasundram SK, Vadamalai G, Pradhan B (2019). Estimating chlorophyll content at leaf scale in viroid-inoculated oil palm seedlings (Elaeis guineensis Jacq) using reflectance spectra (400 nm–1050 nm). Int J Remote Sens.

[38] Zarco-Tejada PJ, Hornero A, Beck PSA, Kattenborn T, Kempeneers P, Hernández-Clemente R (2019). Chlorophyll content estimation in an open-canopy conifer forest with Sentinel-2A and hyperspectral imagery in the context of forest decline. Remote Sens Environ.

[39] Zhou X, Huang W, Zhang J, Kong W, Casa R, Huang Y (2019). A novel combined spectral index for estimating the ratio of carotenoid to chlorophyll content to monitor crop physiological and phenological status. Int J Appl Earth Obs Geoinf.

[40] Laurent VCE, Schaepman ME, Verhoef W, Weyermann J, Chávez RO (2014). Bayesian object-based estimation of LAI and chlorophyll from a simulated Sentinel-2 top-of-atmosphere radiance image. Remote Sens Environ.

[41] Zou X, Hernández-Clemente R, Tammeorg P, Lizarazo Torres C, Stoddard FL, Mäkelä P (2015). Retrieval of leaf chlorophyll content in field crops using narrow-band indices: effects of leaf area index and leaf mean tilt angle. Int J Remote Sens.

[42] Simic Milas A, Romanko M, Reil P, Abeysinghe T, Marambe A (2018). The importance of leaf area index in mapping chlorophyll content of corn under different agricultural treatments using UAV images. Int J Remote Sens.

[43] Li Z, Jin X, Wang J, Yang G, Nie C, Xu X (2015). Estimating winter wheat (Triticum aestivum) LAI and leaf chlorophyll content from canopy reflectance data by integrating agronomic prior knowledge with the PROSAIL model. Int J Remote Sens.

[44] Croft H, Chen JM, Zhang Y (2014). Temporal disparity in leaf chlorophyll content and leaf area index across a growing season in a temperate deciduous forest. Int J Appl Earth Obs Geoinf.

[45] Jay S, Gorretta N, Morel J, Maupas F, Bendoula R, Rabatel G (2017). Estimating leaf chlorophyll content in sugar beet canopies using millimeter- to centimeter-scale reflectance imagery. Remote Sens Environ.

[46] Malenovsky Z, Ufer C, Lhotáková Z, Clevers JG, Schaepman ME, Albrechtová J, Cudlín P (2006). A new hyperspectral index for chlorophyll estimation of a forest canopy: area under curve normalised to maximal band depth between 650-725 nm. EARSeL eProceedings.

[47] Sykioti O, Paronis D, Stagakis S, Kyparissis A (2011). Band depth analysis of CHRIS/PROBA data for the study of a Mediterranean natural ecosystem Correlations with leaf optical properties and ecophysiological parameters. Remote Sens Environ.

[48] He R, Li H, Qiao X, Jiang J (2018). Using wavelet analysis of hyperspectral remote-sensing data to estimate canopy chlorophyll content of winter wheat under stripe rust stress. Int J Remote Sens.

[49] Li D, Cheng T, Zhou K, Zheng H, Yao X, Tian Y (2017). WREP: a wavelet-based technique for extracting the red edge position from reflectance spectra for estimating leaf and canopy chlorophyll contents of cereal crops. ISPRS J Photogramm Remote Sens..

[50] Gitelson AA, Gritz Y, Merzlyak MN (2003). Relationships between leaf chlorophyll content and spectral reflectance and algorithms for non-destructive chlorophyll assessment in higher plant leaves. J Plant Physiol.

[51] Haboudane D, Miller JR, Tremblay N, Zarco-Tejada PJ, Dextraze L (2002). Integrated narrow-band vegetation indices for prediction of crop chlorophyll content for application to precision agriculture. Remote Sens Environ.

[52] Zarco-Tejada PJ, Hornero A, Hernández-Clemente R, Beck PSA (2018). Understanding the temporal dimension of the red-edge spectral region for forest decline detection using high-resolution hyperspectral and Sentinel-2a imagery. ISPRS J Photogramm Remote Sens..

[53] Wu C, Niu Z, Tang Q, Huang W (2008). Estimating chlorophyll content from hyperspectral vegetation indices: modeling and validation. Agric For Meteorol.

[54] Gitelson A, Merzlyak MN (1994). Spectral reflectance changes associated with autumn senescence of *Aesculus hippocastanum* L. and *Acer platanoides* L. leaves spectral features and relation to chlorophyll estimation. J Plant Physiol.

[55] Daughtry CST, Walthall CL, Kim MS, De Colstoun EB, McMurtrey JE (2000). Estimating corn leaf chlorophyll concentration from leaf and canopy reflectance. Remote Sens Environ.

[56] Schlemmera M, Gitelson A, Schepersa J, Fergusona R, Peng Y, Shanahana J (2013). Remote estimation of nitrogen and chlorophyll contents in maize at leaf and canopy levels. Int J Appl Earth Obs Geoinf.

[57] Barnes EM, Clarke TR, Richards SE, Colaizzi PD, Haberland J, Kostrzewski M, et al. Coincident detection of crop water stress, nitrogen status and canopy density using ground based multispectral data. Proc 5th Int Conf Precis Agric. 2000. https://www.tucson.ars.ag.gov/unit/Publications/PDFfiles/1356.pdf.

[58] Lu S, Lu F, You W, Wang Z, Liu Y, Omasa K (2018). A robust vegetation index for remotely assessing chlorophyll content of dorsiventral leaves across several species in different seasons. Plant Methods..

[59] Li Y, Sun Y, Jiang J, Liu J (2019). Spectroscopic determination of leaf chlorophyll content and color for genetic selection on Sassafras tzumu. Plant Methods..

[60] Goulas Y, Cerovic ZG, Cartelat A, Moya I (2004). Dualex: a new instrument for field measurements of epidermal ultraviolet absorbance by chlorophyll fluorescence. Appl Opt.

[61] Yang G, Li C, Wang Y, Yuan H, Feng H, Xu B (2017). The DOM generation and precise radiometric calibration of a UAV-mounted miniature snapshot hyperspectral imager. Remote Sens..

[62] Parry C, Blonquist JM, Bugbee B (2014). In situ measurement of leaf chlorophyll concentration: analysis of the optical/absolute relationship. Plant Cell Environ.

[63] Padilla FM, Teresa Peña-Fleitas M, Gallardo M, Thompson RB (2014). Evaluation of optical sensor measurements of canopy reflectance and of leaf flavonols and chlorophyll contents to assess crop nitrogen status of muskmelon. Eur J Agron.

[64] Cerovic ZG, Masdoumier G, Ghozlen NB, Latouche G (2012). A new optical leaf-clip meter for simultaneous non-destructive assessment of leaf chlorophyll and epidermal flavonoids. Physiol Plant.

[65] Verhoef W (1984). Light scattering by leaf layers with application to canopy reflectance modeling: the SAIL model. Remote Sens Environ.

[66] Gonsamo A, Chen JM (2013). Spectral response function comparability among 21 satellite sensors for vegetation monitoring. IEEE Trans Geosci Remote Sens.

[67] Trishchenko AP, Cihlar J, Li Z (2002). Effects of spectral response function on surface reflectance and NDVI measured with moderate resolution satellite sensors. Remote Sens Environ.

[68] Trishchenko AP (2009). Effects of spectral response function on surface reflectance and NDVI measured with moderate resolution satellite sensors: extension to AVHRR NOAA-17, 18 and METOP-A. Remote Sens Environ.

[69] Rouse et al. Monitoring vegetation systems in the great plains with ERTS. Third Earth Resour Technol Satell Symp. 1973;1:309–17. https://ntrs.nasa.gov/archive/nasa/casi.ntrs.nasa.gov/19740022614.pdf.

[70] Rondeaux G, Steven M, Baret F (1996). Optimization of soil-adjusted vegetation indices. Remote Sens Environ.

[71] Huete A, Didan K, Miura T, Rodriguez EP, Gao X, Ferreira LG (2002). Overview of the radiometric and biophysical performance of the MODIS vegetation indices. Remote Sens Environ.

[72] Jiang Z, Huete AR, Didan K, Miura T (2008). Development of a two-band enhanced vegetation index without a blue band. Remote Sens Environ.

[73] Roujean JL, Breon FM (1995). Estimating PAR absorbed by vegetation from bidirectional reflectance measurements. Remote Sens Environ.

