# Supplementary material for: A robust spectral angle index for remotely assessing soybean canopy chlorophyll content in different growing stages
Source: Plant Methods. 2020 Jul 31;16:104. doi: 10.1186/s13007-020-00643-z (PMC7395406; doi:10.1186/s13007-020-00643-z)
Supplement: Supplementary file 1 — Additional file 1. Additional table and figures. [file 13007_2020_643_MOESM1_ESM.docx]

**Supplementary information:**

Table S1 shows the spectral bands for the Sentinel-2 MSI sensors. Figure S1 shows the Sentinel-2 MSI SRF in optical bands (B2, B3, B4, B5, B6, B7, and B8, see Table S1). Figure S2 shows the field measured and PROSAIL-based LAI vs Chl.

**Table S1.** Spectral bands for the Sentinel-2 MSI sensors. Note: From https://earth.esa.int/web/sentinel/technical-guides/sentinel-2-msi/msi-instrument.

| **Bands** | **Band names/abbreviations** | **Sentinel-2A** | | **Sentinel-2B** | | **Spatial**  **resolution**  **(m)** |
| --- | --- | --- | --- | --- | --- | --- |
|  |  | **Central**  **wavelength**  **(nm)** | **Band**  **width**  **(nm)** | **Central**  **wavelength**  **(nm)** | **Band**  **width**  **(nm)** |  |
| 1 | - | 442.7 | 21 | 442.3 | 21 | 60 |
| 2 | Blue (B) | 492.4 | 66 | 492.1 | 66 | 10 |
| 3 | Green (G) | 559.8 | 36 | 559 | 36 | 10 |
| 4 | Red (R) | 664.6 | 31 | 665 | 31 | 10 |
| 5 | Red edge1 (RE1) | 704.1 | 15 | 703.8 | 16 | 20 |
| 6 | Red edge2 (RE2) | 740.5 | 15 | 739.1 | 15 | 20 |
| 7 | Red edge3 (RE3) | 782.8 | 20 | 779.7 | 20 | 20 |
| 8 | Near-infrared (NIR) | 832.8 | 106 | 833 | 106 | 10 |
| 8a | - | 864.7 | 21 | 864 | 22 | 20 |
| 9 | - | 945.1 | 20 | 943.2 | 21 | 60 |
| 10 | - | 1373.5 | 31 | 1376.9 | 30 | 60 |
| 11 | - | 1613.7 | 91 | 1610.4 | 94 | 20 |
| 12 | - | 2202.4 | 175 | 2185.7 | 185 | 20 |


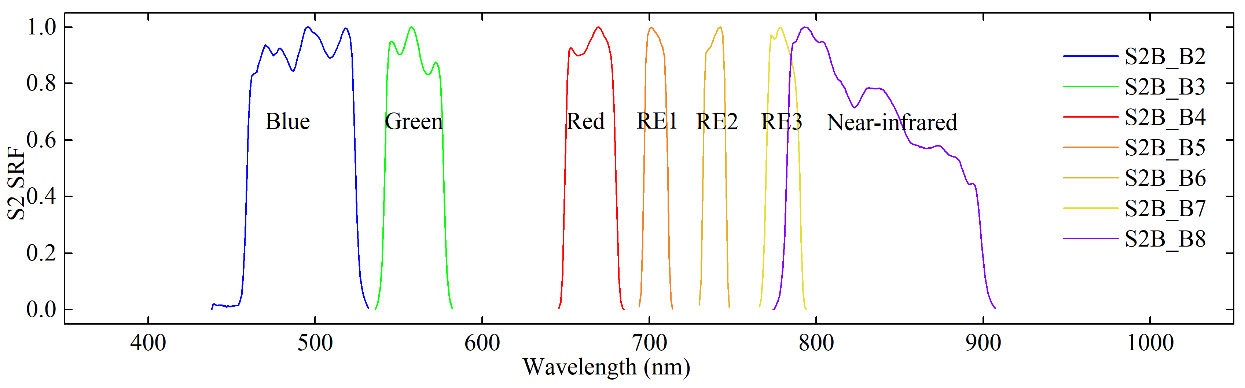


**Fig. S1.** Sentinel-2 MSI SRF in optical bands (B2, B3, B4, B5, B6, B7, and B8, see Table S1). Note: From https://earth.esa.int/web/sentinel/user-guides/sentinel-2-msi/document-library/-/asset_publisher/Wk0TKajiISaR/content/sentinel-2a-spectral-responses


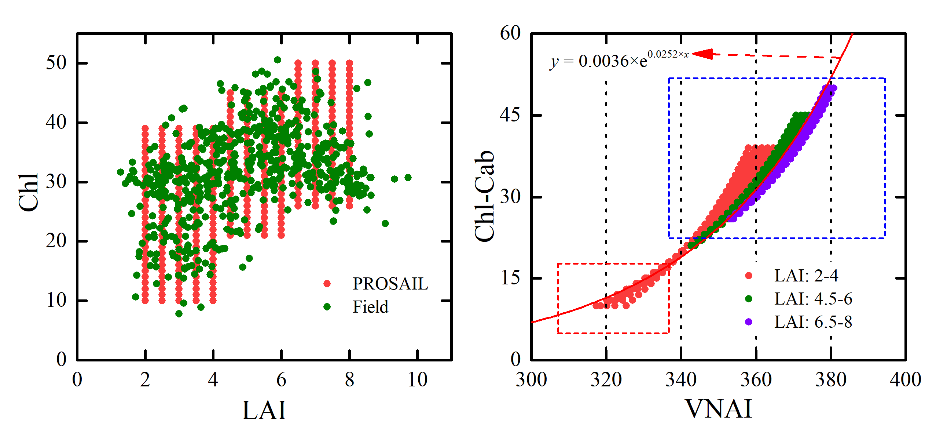


**Fig. S2.** Field measured and PROSAIL-based LAI vs Chl. Note: In this work, the soybean LAI was measured by using LAI2200 sensor (LI-COR, Lincoln, NE, USA). The LAI2200 instrument enables the rapid measurement of crop LAI under field conditions. The measurements were performed using a 90° view caps from 4:00 p.m. to 6:00 p.m. Five measurements (center and four corners) of each soybean plot were collected. After the collection of LAI data, the average values were recorded as the LAI of each soybean plot.
